# Genome-wide annotation, expression profiling, and protein interaction studies of the core cell-cycle genes in *Phalaenopsis aphrodite*

**DOI:** 10.1007/s11103-013-0128-y

**Published:** 2013-09-25

**Authors:** Hsiang-Yin Lin, Jhun-Chen Chen, Miao-Ju Wei, Yi-Chen Lien, Huang-Hsien Li, Swee-Suak Ko, Zin-Huang Liu, Su-Chiung Fang

**Affiliations:** 1 Biotechnology Center in Southern Taiwan, Academia Sinica, No. 59, Siraya Blvd., Xinshi District, Tainan, 741 Taiwan; 2 Agricultural Biotechnology Research Center, Academia Sinica, Taipei, 115 Taiwan; 3 Institute of Tropical Plant Sciences, National Cheng Kung University, Tainan, 701 Taiwan

**Keywords:** Cell cycle, *Phalaenopsis aphrodite*, Orchid, Protocorm, Meristematic tissues

## Abstract

Orchidaceae is one of the most abundant and diverse families in the plant kingdom and its unique developmental patterns have drawn the attention of many evolutionary biologists. Particular areas of interest have included the co-evolution of pollinators and distinct floral structures, and symbiotic relationships with mycorrhizal flora. However, comprehensive studies to decipher the molecular basis of growth and development in orchids remain scarce. Cell proliferation governed by cell-cycle regulation is fundamental to growth and development of the plant body. We took advantage of recently released transcriptome information to systematically isolate and annotate the core cell-cycle regulators in the moth orchid *Phalaenopsis aphrodite*. Our data verified that *Phalaenopsis* cyclin-dependent kinase A (CDKA) is an evolutionarily conserved CDK. Expression profiling studies suggested that core cell-cycle genes functioning during the G1/S, S, and G2/M stages were preferentially enriched in the meristematic tissues that have high proliferation activity. In addition, subcellular localization and pairwise interaction analyses of various combinations of CDKs and cyclins, and of E2 promoter-binding factors and dimerization partners confirmed interactions of the functional units. Furthermore, our data showed that expression of the core cell-cycle genes was coordinately regulated during pollination-induced reproductive development. The data obtained establish a fundamental framework for study of the cell-cycle machinery in *Phalaenopsis* orchids.

**Electronic supplementary material:**

The online version of this article (doi:10.1007/s11103-013-0128-y) contains supplementary material, which is available to authorized users.

## Introduction

Cell reproduction is a basic biological process that is essential for the growth of all organisms (Hall et al. 2004). Cell-cycle regulation plays a pivotal role in the cell proliferation required for plant growth. The core cell-cycle genes are central regulators of cell division and hence a common converging point for internal and external signals during growth and development. In plants, growth derives from meristematic tissues. Cell proliferation regulated by intrinsic developmental signals and extrinsic environmental cues is required for meristem replenishment and organization. In addition, cell division machinery governing cell proliferation has to be carefully regulated in order to meet differentiation and fate specification requirements during plant development.

The eukaryotic cell cycle is controlled by an evolutionarily conserved set of cell-cycle proteins (Mironov et al. 1999). Despite the universal principle of cell-cycle regulation, the core cell-cycle genes in plants are duplicated and have diverged to accommodate complex developmental requirements. Based on a homology-based annotation strategy, more than 90 cell-cycle genes have been classified in Arabidopsis (Vandepoele et al. 2002; Menges et al. 2005). However, how these cell-cycle proteins are coordinated to drive cell division and regulate differentiation programs in plants remains largely unclear.

Cyclin-dependent kinases (CDKs) are central cell-cycle regulators whose activities are subject to multiple levels of regulation and whose expression oscillates in a periodic manner to drive cell-cycle phase transitions (Morgan 2007). Based on the nomenclature derived from genome-wide classification of the Arabidopsis cell-cycle genes, there are eight classes of CDKs, designated as A to G type and CDK-like (L-type) CDKs (Vandepoele et al. 2002; Menges et al. 2005). CDK activity is activated by binding to cyclins (CYCs), inhibited by CDK inhibitors, and regulated by phosphorylation. Plant cell-cycle transitions are controlled by two types of CDKs—A-type CDKs (CDKAs) and B-type CDKs (CDKBs). Plant CDKA contains a canonical cyclin-binding motif PSTAIRE in the alpha-1 helix that is conserved across different kingdoms (Inze and De Veylder 2006). CDKA has been shown to play a pivotal role at both the G1/S and G2/M transitions and can functionally rescue CDK-deficient yeast mutants (Ferreira et al. 1991; Hirayama et al. 1991). Members of the CDKB gene family are plant specific (Burssens et al. 1998; Mironov et al. 1999). They have been reported to play roles during the G2-M and S-G2-M phases (Fobert et al. 1996; Magyar et al. 1997; Umeda et al. 1999; Meszaros et al. 2000; Porceddu et al. 2001; Sorrell et al. 2001; Breyne et al. 2002; Menges et al. 2002; Corellou et al. 2005).

Cyclins activate the kinase activity of the CDKs and control timely entry into the cell cycle. In plants and animals, three main classes of CYCs are required for cell-cycle progression. Generally, but not exclusively, D-type CYCs function in the G1 stage and are required to regulate G1/S transition, A-type CYCs function during S phase, and B-type CYCs are important in regulation of S phase and mitosis. Transcripts of CDKB and CYCB gene family members are preferentially accumulated during S, G2, and M phases and are often used as markers for dividing cells (Inze and De Veylder 2006). Plant A- and B-type CYCs have orthologs in animals. Plant D-type CYCs show less similarity to D-type CYCs from animals and are therefore referred to as plant specific (Wang et al. 2004). There are at least 30 CYCs with potential roles in cell-cycle regulation in Arabidopsis (Vandepoele et al. 2002). Because D-type CYCs regulate G1-S transition and the commitment to enter the cell cycle, they are thought to be primary targets for external and internal signals leading to cell proliferation (Nieuwland et al. 2009). However, many exceptions to this simple functional assignment have been reported (Inze and De Veylder 2006; Vanneste et al. 2011). Therefore, the functional role of individual CYCs may be oversimplified and their roles during cell-cycle progression need to be tested experimentally.

Cyclin-dependent kinase activity is also modulated by CDK-activating kinases (CAKs). Two types of CAKs have been found in higher plants that have been designated as D- and F-type. *CDKDs* are functionally related to vertebrate CAKs, whereas *CDKFs* are plant specific. D- and F-type CDKs have been shown to activate A-type CDKs via phosphorylation (Umeda et al. 1998; Yamaguchi et al. 2000; Shimotohno et al. 2003). In Arabidopsis, cyclin H-activated CDKDs are capable of phosphorylating CDKs as well as the C-terminal domain of the largest subunit of RNA polymerase II (Shimotohno et al. 2004). Conversely, F-type CDKs do not require a CYC interacting partner and are capable of phosphorylating CDKs (Shimotohno et al. 2004, 2006). Arabidopsis *CDKF* has been shown to phosphorylate and activate CDKD (Shimotohno et al. 2004).

Cyclin-dependent kinase inhibitors (CKIs) negatively regulate the CDK activity by direct binding. Plants contain CKIs that show weak similarity to the N-terminally located CKI domain of the mammalian Cip/Kip proteins (Wang et al. 1997; Lui et al. 2000) and are, therefore, commonly referred to as Kip-related proteins (KRPs). The Arabidopsis KRP protein family has seven members. Work with Arabidopsis has shown that the KRP family proteins are expressed in both mitotically dividing and endoreduplicating cells (Ormenese et al. 2004). The involvement of some *KRP* genes in regulating endoreduplication (part of the plant differentiation program) has been confirmed by overexpression approaches. For instance, overexpression of either Arabidopsis *KRP1* or *KRP2,* preferentially expressed in endoreduplicating cells, reduces cell division and affects the switch to the endoreduplication cycle (Wang et al. 2000; De Veylder et al. 2001; Verkest et al. 2005b; Gutierrez 2005; De Veylder et al. 2007; Verkest et al. 2005a). The defects caused by overexpression of KRP1 are overcome by simultaneous overexpression of D-type CYCs (Jasinski et al. 2002; Schnittger et al. 2003; Zhou et al. 2003). Additionally, *KRP6* whose expression is present in both mitotically dividing and endoreduplicating cells (Ormenese et al. 2004) is required for timely regulation of cell-cycle progression during gametogenesis (Liu et al. 2008).

In yeast and animal systems, the kinase activity of the CDKs is negatively regulated by the WEE1 family kinases (Kellogg 2003; Perry and Kornbluth 2007). WEE1 protein kinases phosphorylate a conserved Tyr residue of the CDKs and negatively regulate CDK activity. Such negative regulation is necessary to coordinate transition between DNA replication and mitosis (Russell and Nurse 1987; Gould and Nurse 1989; Jin et al. 1996). In Arabidopsis, *WEE1* transcripts are induced under DNA damage conditions. WEE1 deficient plants grow normally without obvious cell division or endoreduplication phenotype but display hypersensitivity to DNA-damaging agents (De Schutter et al. 2007).

The retinoblastoma (RB)/E2 promoter-binding factors (E2F)/dimerization partners (DP) pathway plays a pivotal role in control of G1/S transition in eukaryotic organisms (Weinberg 1995; Gutierrez 1998, 2005). Within this paradigm, RB protein represses transcription of E2F-regulated genes by physically interacting with the heterodimeric E2F/DP transcription factor complex (Harbour and Dean 2000). The mitogenic signal induces cyclin-activated CDKA activity, which in turn phosphorylates RB and releases RB from E2F/DP promoter complexes. Active transcriptional activity of E2F/DP protein then allows expression of S-phase genes and cell cycle entry. Like animal Rb, plant retinoblastoma-related (RBR) protein is phosphorylated by CYCD-activated CDKA activity in a cell cycle-dependent manner (Boniotti and Gutierrez 2001; Nakagami et al. 2002; Koroleva et al. 2004). However, the biological consequences of RBR phosphorylation/dephosphorylation in regulating cell proliferation and fate specification during plant development remains elusive. In Arabidopsis, *RBR* knock-out mutants are sterile because of excess mitotic divisions in the mature female megagametophyte (Ebel et al. 2004). RBR is also required to regulate differentiation in root stem cells (Wildwater et al. 2005). Reduction of RBR protein negatively influences the establishment of cell differentiation. For instance, meristematic stem cells such as shoot apical meristem cells, meristemoid mother cells, and procambial cells fail to produce differentiated cells subsequently resulting in defects in lateral organ formation (Borghi et al. 2010). In addition, RBR is required for developmental phase transition (Gutzat et al. 2011) and asymmetric stem cell division in roots (Cruz-Ramirez et al. 2012). In summary, RBR is important in the regulation of cell division and differentiation during different stages of plant development.

Plants encode multiple E2F and DP family members (Gutierrez et al. 2002; De Veylder et al. 2002; De Veylder et al. 2003; Dewitte and Murray 2003). E2F/DP protein complexes can serve as transcription activators or repressors (Muller and Helin 2000; Bracken et al. 2004; Gutierrez 2005). In Arabidopsis, overexpression of *AtE2Fa* and *AtE2Fb* with *AtDPa* induced cell proliferation in differentiated tissues (De Veylder et al. 2002; Rossignol et al. 2002; Magyar et al. 2005; Sozzani et al. 2006). Recent work revealed that AtE2Fa stimulates cell proliferation by forming a stable complex with AtRBR1 protein to inhibit endoreduplication and premature differentiation (Magyar et al. 2012). *E2Fc* and *DPb*, on the other hand, work together to exert a negative effect on cell proliferation (del Pozo et al. 2002). In addition to the typical E2F transcription factors, plants have evolved atypical E2F or DP-E2F-like (DEL) factors that lack an RBR-binding motif and possess two DNA-binding domains that allow them to bind as a monomer in a DP-independent manner to E2F target genes (Mariconti et al. 2002; Lammens et al. 2009). These atypical E2F proteins do not contain a transactivation domain and play versatile roles during plant development.

The Orchidaceae family is one of the largest families of flowering plants. Orchid species inhabit a wide range of ecological environments and possess highly specialized morphological, physiological, and developmental characteristics (Dresser 1990). For example, ovule development does not initiate until pollination occurs (Nadeau et al. 1996). Fertilization only occurs after ovules and female gametophytes are fully developed, which normally takes approximately 60–70 days. Additionally, orchid embryos do not have the obvious organized domains for specification of the future organs. During germination, orchid seeds pass through a transitional stage during which a specialized small spherical tuber-like structure termed the protocorm is produced. The protocorm has a meristematic domain at the anterior end where new leaves and roots are derived (Nishimura 1981). Given such unique developmental programs, cell-cycle regulators in orchids may have evolved and be regulated differently to those of other plants to accommodate specialized and unique developmental decisions.

Although many studies have reported the isolation and molecular functions of the core cell-cycle genes in Arabidopsis and rice, similar effort has not been paid to non-model plant species with specialized developmental programs. Despite the general principle of cell-cycle regulation, there are important variations in how cell-cycle programs are modified to deal with environmental cues or developmental decisions across plant species. For instance, endoreduplication (the modified cell-cycle program) is incorporated into DNA stress adaptation in Arabidopsis (Adachi et al. 2011). Rice plants, on the other hand, deal with DNA stress by reducing endoreduplication (Endo et al. 2011). Hence, biological consequences derived from functional studies of the cell-cycle regulators in Arabidopsis might not directly translate to the functions of orthologous counterparts in other plants. Identification and expression profiling analysis of the cell-cycle genes in other species might provide novel insights into diversification of the cell-cycle program across the plant kingdom.

In this study, we conducted a genome-wide study of the core cell-cycle genes of the moth orchid *Phalaenopsis aphrodite* and profiled their expression patterns. We chose *P. aphrodite* because it is an important parent plant for commercial breeding programs in Taiwan, and its transcriptome database is publicly available (Su et al. 2011; An et al. 2011; Fu et al. 2011). We present a comprehensive analysis of transcriptional regulation of the core cell-cycle genes during orchid development. We used yeast two-hybrid (Y2H) and bimolecular fluorescence complementation (BiFC) assays to confirm the interaction network of the selected cell-cycle genes. Furthermore, a protein–protein interaction map provides molecular evidence of the functional units of cell-cycle protein complexes. Taken together, our data represent the first comprehensive characterization of the core cell-cycle genes in the *Phalaenopsis* orchid. In addition, distinct expression patterns of cell-cycle regulators during reproductive development have been documented.

## Materials and methods

### Plant materials

*Phalaenopsis aphrodite* Subsp. *formosana* (v1656, v1644 or v1642) seedlings in 2.5 in. pots were purchased from Chain Port Orchid Nursery (Ping Tung, Taiwan). All plants were grown in alternating 12 h light (23 °C)/12 h dark (18 °C) cycles in a growth chamber with regular irrigation and fertilization.

### Annotation strategy

Comprehensive searches for core cell-cycle genes including CDKs, CYCs, RB-related, E2Fs and DPs, WEE1, and CKIs were conducted on the Orchidstra *Phalaenopsis* Genome Annotation Database (Su et al. 2011). We used the transcripts of core cell-cycle genes from either Arabidopsis or rice (Supplementary Table S1) to run genome-wide Basic Local Alignment Search Tool (BLAST) searches against Orchidstra *Phalaenopsis* Genome Annotation Database http://orchidstra.abrc.sinica.edu.tw/none/. The initial search yielded a few high-scoring positives (E-value of 1e-005 was set as the cutoff). The sequences of tentative EST contigs were retrieved and blasted against the National Center for Biotechnology Information (NCBI) non-redundant protein database to confirm their identification, and against the *Phalaenopsis* Genome Annotation Database to identify additional family members. To categorize gene members from the same family, the BLAST search was repeated with the *Phalaenopsis* candidate genes against the protein database at The Arabidopsis Information Resource and The Michigan State University Rice Genome Annotation Project Database and Resource. The signature motifs conserved during evolution were taken into consideration during annotation. To correct errors of assembled EST contigs and obtain full-length gene models, rapid amplification of cDNA ends (RACE)-PCR and reverse transcription were carried out to confirm and complete the gene models. Based on this strategy, we were able to identify and isolate most of the core cell-cycle genes in *P. aphrodite*. The results from individual gene families are discussed below. The annotation results are summarized in Supplementary Table S2.

### Phylogenetic tree construction

Protein sequences were aligned by ClustalW. The resulting alignments were used to construct phylogenetic trees in Mega 5.05 (Tamura et al. 2011). The Maximum-likelihood method was used to generate phylogenetic trees and 1,000 replicates were used for bootstrapping. Bootstrap values of 50 % or higher were shown for each clade. The evolutionary distances were computed using the Poisson correction method and the rate variation among sites was modeled with a gamma distribution. Gene identification numbers used to generate phylogenetic trees are listed in Supplementary Table S3.

### Yeast two-hybrid assay

cDNA of *PaDP1* or *PaDP2* gene was introduced into pDEST32 vector and an in-frame fusion to the *GAL4* DNA binding domain was generated as bait. cDNA of *PaE2F3* gene was introduced into pDEST22 vector and an in-frame fusion to the *GAL4* activation domain was generated as a prey (Invitrogen, USA). cDNAs of the *PaE2F1* and *PaE2F2* failed to be cloned into the Gateway system. Alternatively, they were introduced into a pGADT7 vector (Clontech, USA) to make an in-frame fusion to the *GAL4* activation domain. cDNA of *PaDP1* or *PaDP2* was cloned into pGBKT7 vector (Clontech, USA) to make an in-frame fusion to the *GAL4* DNA binding domain. The pairs of constructs to be tested were co-transformed into AH109 yeast competent cells using a lithium acetate method according to the manufacturer’s instructions (Invitrogen, USA). Co-transformed yeast cells were selected on plates with Leu (for pDEST32 and pGADT7 plasmids) and Trp (for pDEST22 and pGBKT7 plasmids) drop-out medium for 3–8 days at 30 °C. Transformants were tested for specific interactions by growing on SC–Leu–Trp–His plates with 10 or 20 mM 3-amino-1,2,4 triazole (3AT).

### Complementation of a *S. cerevisiaecdc28*-*as1* mutant

*PaCDKA1* cDNA was cloned into a pYES-DEST52 vector (Invitrogen, USA). The construct was transformed into the *cdc28*-*as1* mutant by the lithium acetate method described above. The transformants were allowed to grow in the presence of 1,000 nM 4-Amino-1-tert-butyl-3-(1′-naphthylmethyl) pyrazolo [3,4-d] pyrimidine (1NM-PP1), Merck Millipore Chemicals, USA) and scored for viability.

### Sample collection and RNA extraction

For gametophytic and embryonic tissues, orchid flowers were hand pollinated and developing ovaries were harvested at the specified day. Only the interior tissues from developing ovaries were scooped and pooled for RNA extraction. For root samples, 2-cm tip tissue containing root apical meristems was collected. For stalk samples, 5–10 cm long stalks were collected. For protocorm-like body (PLB) samples, one-month-old tissues were collected. For protocorm samples, 20- and 30-day-old tissues were pooled and collected. The collected samples were flash frozen in liquid nitrogen and stored in a freezer at −80 °C. RNA was isolated using MaestroZol RNA Plus extraction reagent (Maestrogen, USA) according to the manufacturer’s instructions. The isolated total RNA was treated with RNase-free DNase (Qiagen, USA) followed by RNeasy mini-column purification according to the manufacturer’s instructions (Qiagen, USA).

### Real-time quantitative RT-PCR and RACE-PCR

Three micrograms (for experiments shown in Fig. 5) or 5 μg (for experiments shown in Fig. 9) total RNA was used for cDNA synthesis. DNA-free RNA was reverse transcribed in the presence of a mixture of oligo dT and random primers (9:1 ratio) using the GoScript Reverse Transcription System (Promega, USA) according to the manufacturer’s instructions. Ten microliters of RT-PCR reaction contained 2.5 μl of 1/20 diluted cDNA, 0.2 mM (for experiments shown in Fig. 5) or 0.25 mM (for experiments shown in Fig. 9) of primers, and 5 μl of 2× KAPA SYBR FAST master mix (KAPA Biosystems, USA). The following program was used for amplification: 95 °C for 1 min, 40 cycles of 95 °C for 5 s and 58 °C for 20 s. PCR was performed in triplicate, and the experiments were repeated twice with RNA isolated from independent samples. For *PaCYCA3;1* and *PaCYCD5;4*, annealing and extension temperature was 60 °C. Real-time PCR was performed using a CFX96 Real-Time PCR detection system (Bio-Rad, USA). Quantification analysis was carried out by CFX Manager Software following manufacturer’s instructions (BioRad, USA). Primers used for qPCR are listed in Supplementary Table S4. 5′ and 3′ RACE PCR were carried out using a SMARTer™ RACE cDNA amplification kit according to manufacturer’s instructions (Clontech, USA). Primers used for RACE-PCR are listed in Supplementary Table S5.

### In situ hybridization

Twenty-eight-day old protocorms, female gametophytes, and developing embryos were collected and fixed immediately in 4 % paraformaldehyde, 4 % dimethylsulfoxide, 0.25 % glutaraldehyde, 0.1 % Tween 20, and 0.1 % Triton X-100 in diethylpyrocarbonate-treated H_2_O at 4 °C overnight. Tissues were then dehydrated and infiltrated with Paraplast (Leica, USA) using a KOS Rapid Microwave Labstation (Milestone, USA). Tissues (10 μm thick) were sectioned using a MICROM 315R microtome (Thermo Scientific, USA) and mounted onto a poly-l-lysine-coated slide (Matsunami, Japan). Sections were then de-paraffinized in xylene, rehydrated in decreasing concentrations of ethanol, and digested with 2 mg/ml proteinase K at 37 °C for 30 min. In situ hybridization was performed as previously described (Bi et al. 2005) with slight modification. Briefly, hybridization was performed at 59 °C in the presence of 40 ng of DIG-labeled RNA probe. Sense and antisense probes were synthesized using a SP6/T7 digoxigenin RNA labeling kit according to the manufacturer’s instructions (Roche, USA). Hybridization signals were detected by NBT/BCIP detection kit (Roche, USA). Tissue sections and in situ hybridization were photographed on a Zeiss Axio Scope A1 microscope equipped with an AxioCam HRc camera (Zeiss, Germany).

### BiFC assay and microscopy

cDNAs encoding the CDK genes were introduced into pE3134 [for details, please see http://www.bio.purdue.edu/people/faculty/gelvin/nsf/protocols_vectors.htm (Tzfira et al. 2005; Citovsky et al. 2006)]. The N-terminal (amino acid residues 1–174) half of YFP was C-terminally in-frame fused to the *CDK* protein. cDNAs encoding the CYC genes were introduced into the pE3130 or pE3132 vectors. The C-terminal (amino acid residues 175–239) half of YFP was N-terminally or C-terminally in-frame fused to the CYC proteins (N(cEYFP)-PaCYC or C(cEYFP)-PaCYC). For PACYCB1;2 and PaCYCD2;1, only the N-terminally tagged version, N(cEYFP)-PaCYCB1;2 and N(cEYFP)-PaCYCD2;1, was obtained. Ballistic bombardment-mediated transient transformation was carried out as previously described (Hsu et al. 2011a) with slight modification. Briefly, 1 μg DNA coated on gold particles (1 μm in diameter) was bombarded into white petals of *Phalaenopsis* Soga Yukidian ‘V3’ using a Biolistic PDS-1000-He particle delivery system (Bio-Rad, USA) at the following settings: helium pressure of projectile, 1,100 psi; 27 mmHg partial vacuum; and target-distance, 9 cm. Florescence images were photographed on a LSM 780 Plus ELYRA S.1 Confocal Microscope with Plan-Apochromat 40×/1.4 oil objective lens (Zeiss, Germany).

## Results

### Annotation of core cell-cycle genes in the *P. aphrodite* database

BLAST searches were conducted to identify the major cell-cycle genes in a *P. aphrodite* transcriptome database. Sixty-five potential genes encoding the core cell-cycle proteins important for cell-cycle control were identified. The isolated genes belonged to the CDK, CYC, Rb, E2F/DP, Wee1, and CKI gene families.

#### *CDK* family

**Fig. 1:**
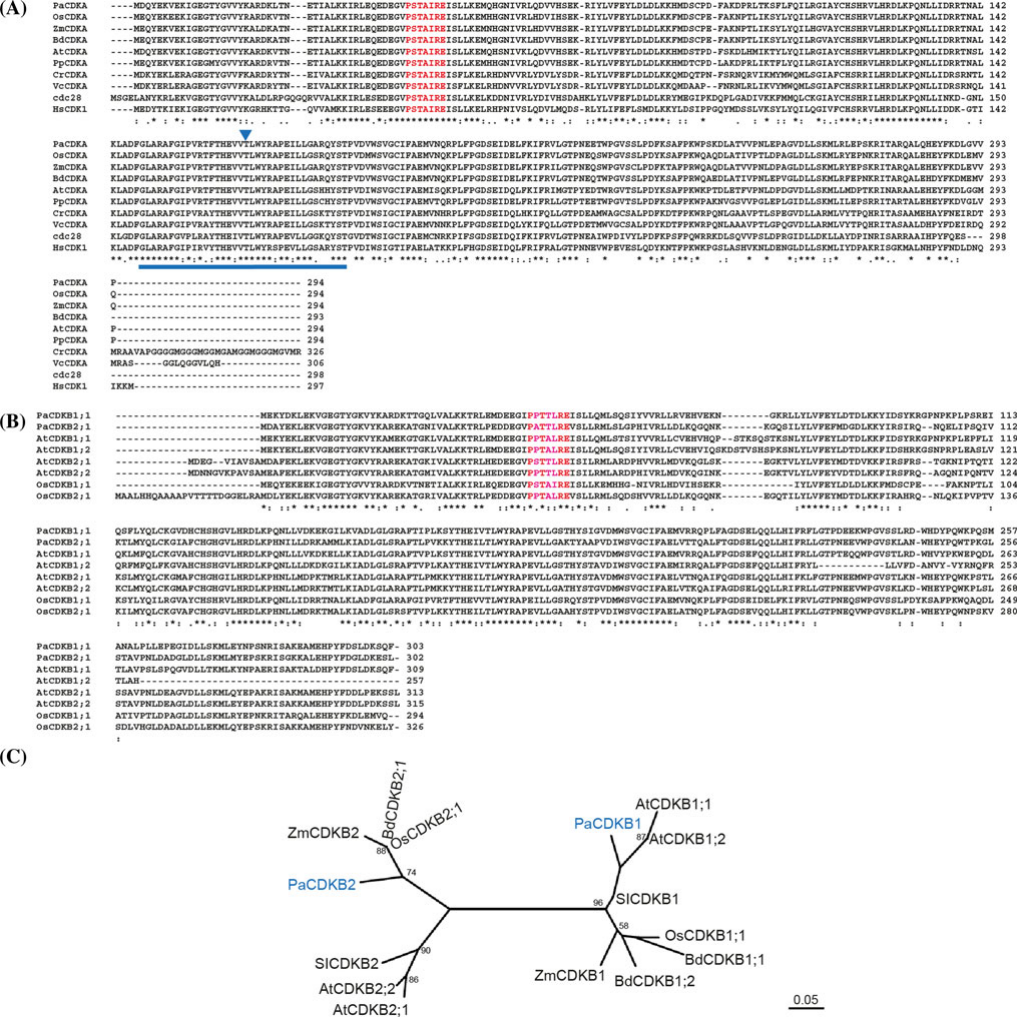
ClustalW alignment of **a** CDKA1 and **b** CDKB proteins. *Asterisk* indicates a conserved residue. *Colon* indicates a residue showing strong similarity among sequences. *Dot* indicates a residue showing weak similarity among sequences. The conserved PSTAIRE motif for cyclin binding among various CDKA1 proteins is marked in *red*. The conserved T-loop of A-type CDK is indicted by a *blue bar*. The conserved phosphorylating threonine residue is labeled in *blue* and marked by an *inverse triangle*. The modified P[P/A/S]T[T/A][I/L]RE motif of B-type CDK proteins in various plant species is marked in *red*. **c** Unrooted maximum-likelihood tree of B-type CDK proteins from different plant species. cdc28, cell division cycle 28; Pa, *P. aphrodite*; Os, *Oryza sativa*; Zm, *Zea mays*; Bd, *Brachypodium distachyon*; At, *Arabidopsis thaliana*; Pp, *P. patens*; Cr, *Chlamydomonas reinhardtii*; Vc, *Volvox carteri*; HS, *Homo sapiens*; Sl, *Solanum lycopersicum*

At least one member of each class of CDK was identified in the *P. aphrodite* genome (Supplementary Table S1). A- and B-type CDKs are the major CDKs that control cell-cycle transitions in plant cells. *P. aphrodite* contains at least one ortholog of the *CDKA* genes, which was designated as *PaCDKA1*. Like other plant CDKA proteins, PaCDKA1 protein has a conserved PSTAIRE signature in the alpha-1 helix, a hallmark CYC-binding motif (Fig. 1a). In addition, the T-loop and the phosphorylating threonine residue required for activation are also conserved in PaCDKA1 (Fig. 1a). The plant *CDKB* family has been classified into two subgroups (Vandepoele et al. 2002; Dewitte and Murray 2003; Inze and De Veylder 2006). The CDKB1 subgroup has PPTALRE as the CYC binding motif. The CDKB2 subgroup is characterized by a P[S/P]TTLRE signature motif. Orthologs of B-type CDKs designated as *PaCDKB1* and *PaCDKB2* were annotated. *PaCDKB1* and *PaCDKB2* share 64 % identity and 81 % similarity at the amino acid level. Based on a previous classification (Dewitte and Murray 2003; Inze and De Veylder 2006; Vandepoele et al. 2002), *PaCDKB1* contains a PPTTLRE motif and therefore belongs to the type 2 CDKB subgroup (Fig. 1b). However, phylogenetic analysis indicated that *PaCDKB1* was clustered with type 1 CDKB members that contain the PPTALRE motif (Fig. 1c). *PaCDKB2,* on the other hand, carried a modified signature motif—PATTLRE—and formed a cluster with a group of monocot CDKB family members (Fig. 1b, c). The PATTLRE motif was also found in CDKB family members of the moss *Physcomitrella patens* (unpublished data). Of the CAKs, at least one member each of the CDKD and CDKF family was identified from the *Phalaenopsis* annotation database (Supplementary Tables S1, S2). The other CDK classes are C-type, E-type, G-type, and CDK-like (CKL) families. At least one gene from each of the CDKC and CDKE families, two CDKG and 13 CDK-like (CKL) genes were identified from the *P. aphrodite* transcriptome database (Supplementary Table S1). Plant C-type CDKs and E-type CDKs are characterized by their similarity to mammalian CDK7 and CDK8, respectively (Joubes et al. 2000; Kitsios et al. 2008). They are assumed to regulate transcription in a similar manner to their counterparts in mammals (Inagaki and Umeda 2011). Arabidopsis CDKC has been shown to be involved in splicing-related transcriptional regulation (Kitsios et al. 2008). Like other plant CDKC protein kinases (Joubes et al. 2000), the CDKC1 of *P. aphrodite* also carries the PITAIRE motif (Supplementary Table S2). The G-type CDK class is homologous to the human p58 galactosyltransferase protein whose role is important for cytokinesis (Menges et al. 2005). Co-purification of CDKGs with CYCL1 suggests that CYCL1 may be an interacting partner of CDKG (Van Leene et al. 2010). CYCL was also identified from the *P. aphrodite* transcriptome database (Supplementary Table S1). The functions of plant CKL protein kinases remain to be clarified. Because A- and B-type CDKs are the only CDKs that directly regulate cell-cycle progression in plants (Vandepoele et al. 2002; Menges et al. 2005), we chose to focus the remainder of our study on A- and B-type CDKs.

#### Cyclin family

**Fig. 2:**
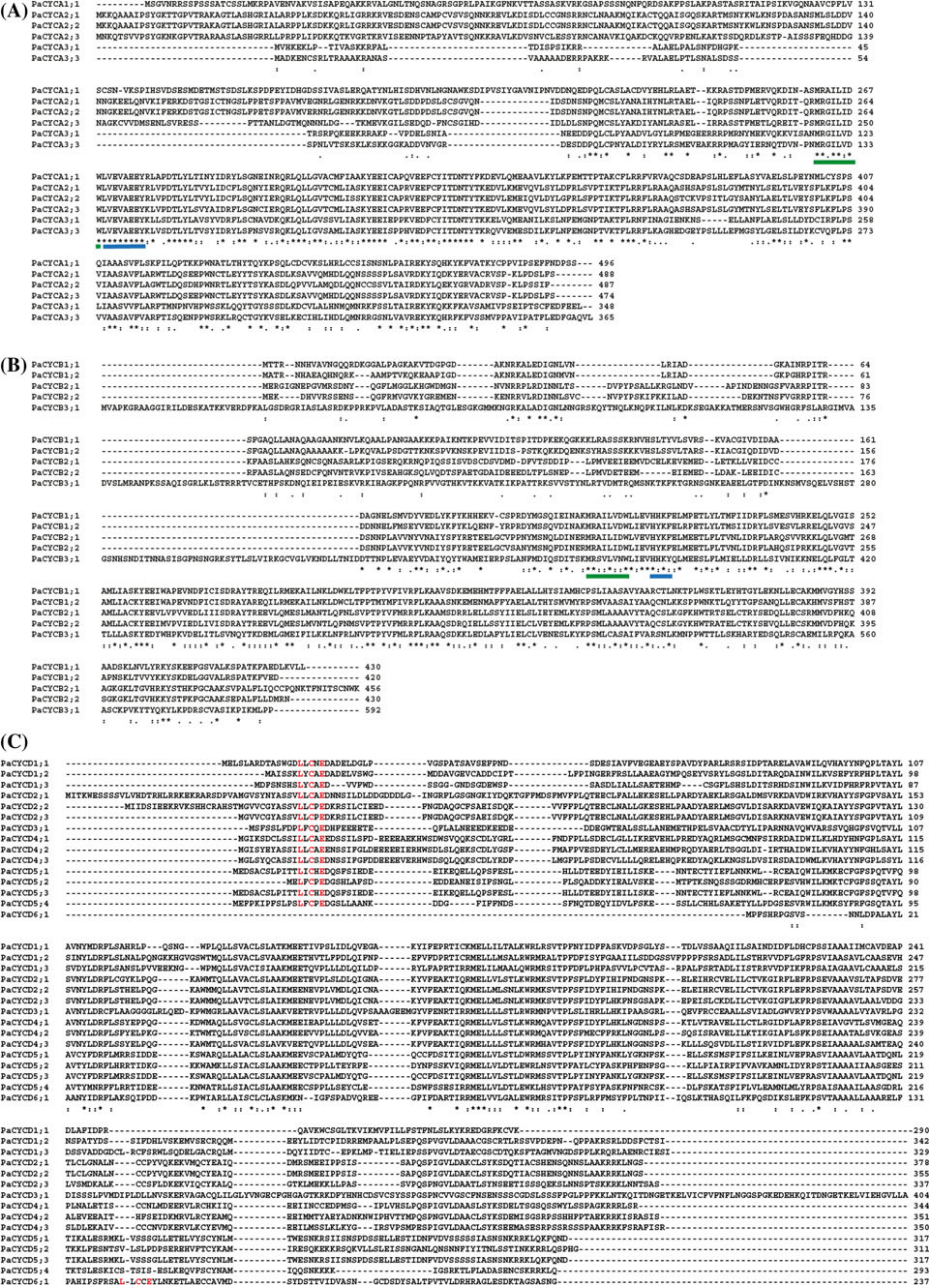

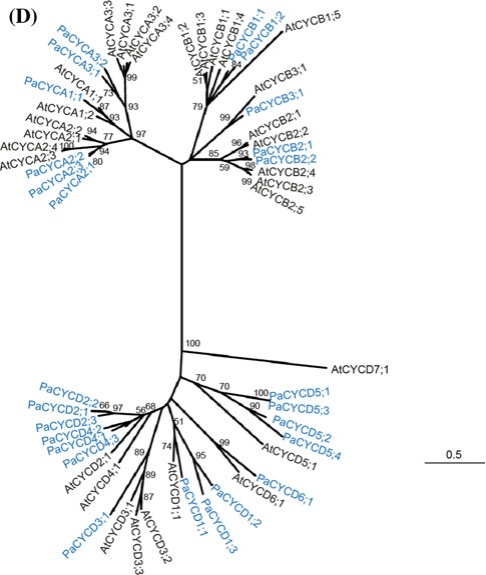
ClustalW alignment of **a** A-type CYCs, **b** B-type CYCs, and **c** D-type CYCs. *Asterisk* indicates a conserved residue. *Colon* indicates a residue showing strong similarity among sequences. *Dot* indicates a residue showing weak similarity among sequences. The A-type CYCs have a conserved LVEVxEEY motif (marked by a *blue bar*) and a CYC box motif (*green bar*). B-type CYCs have a conserved HXKF motif (*blue bar*) and a CYC box motif (*green bar*). The conserved LXCXC motif is marked in *red*. **d** Unrooted maximum-likelihood tree of D-, A-, and B-type CYCs from *P. aphrodite* (Pa) and *A. thaliana* (At). Bootstrap values of 50 % or higher are shown for each clade. ClustalW alignment of *P. aphrodite*

There are 10 types of CYCs in Arabidopsis (Wang et al. 2004), five of which were identified in the *P. aphrodite* transcriptome database (Supplementary Table S2). BLAST analysis revealed that at least six A-type CYCs (CYCAs) belonging to three different subgroups of the family (Chaboute et al. 2000) were present in the *P. aphrodite* transcriptome. They were designated as *PaCYCA1;1*, *PaCYCA2;1*, *PaCYCA2;2*, *PaCYCA2;3*, *PaCYCA3;1*, and *PaCYCA3;2* (Fig. 2a). All *PaCYCA* genes encoded proteins containing a typical LVEVxEEY (x = any amino acid) signature that is shared by A-type CYCs in plant and animal systems (Renaudin et al. 1996) and a conserved CYC box motif MRA/GILI/VDW (Morgan 2007) (Fig. 2a). Five B-type CYCs were identified from our genome-wide analysis (Fig. 2b). Four of them have a CYC B-specific HXKF motif and a conserved CYC box motif: MRAILVDW (for the *PaCYCB1* subgroup), and MRAILIDW (for the *PaCYCB2* subgroup). Fifteen members from six different subgroups of the D-type CYC gene family were annotated. All but CYCD6;1 shared a conserved N-terminal motif LXCXE that was found to bind to the pocket domain of RB-related proteins (Fig. 2c, Supplementary Table S2). PaCYCD6;1, on the other hand, contained a LXCXE motif at the C-terminal end of the protein (Fig. 2c). This suggests that *Phalaenopsis* D-type CYCs are capable of interacting with RB protein to regulate the cell-cycle progression. No *CYCD7* was found in available orchid transcriptome databases (Su et al. 2011; Tsai et al. 2013). To address the lack of the evolutionarily conserved *CYCD7* in this study, we did low-stringency Southern blotting of the *Phalaenopsis* orchid genomic DNA and probed with the rice cDNA fragment corresponding to the conserved regions among monocot *CYCD7;1*. No *Phalaenopsis* *CYCD7* signal was detected (Supplementary Fig. S1). However, we cannot rule out the possibility that the orchid *CYCD7* sequence may be too divergent to be detected by Southern blotting. *Phalaenopsis* D-, A-, and B-type CYCs were clearly separated and formed clades with different CYC groups in Arabidopsis (Fig. 2d). The remaining CYCs identified from the *P. aphrodite* transcriptome were H- or L-type CYCs (Supplementary Table S2).

#### *RB* and *E2F/DP* families

**Fig. 3:**
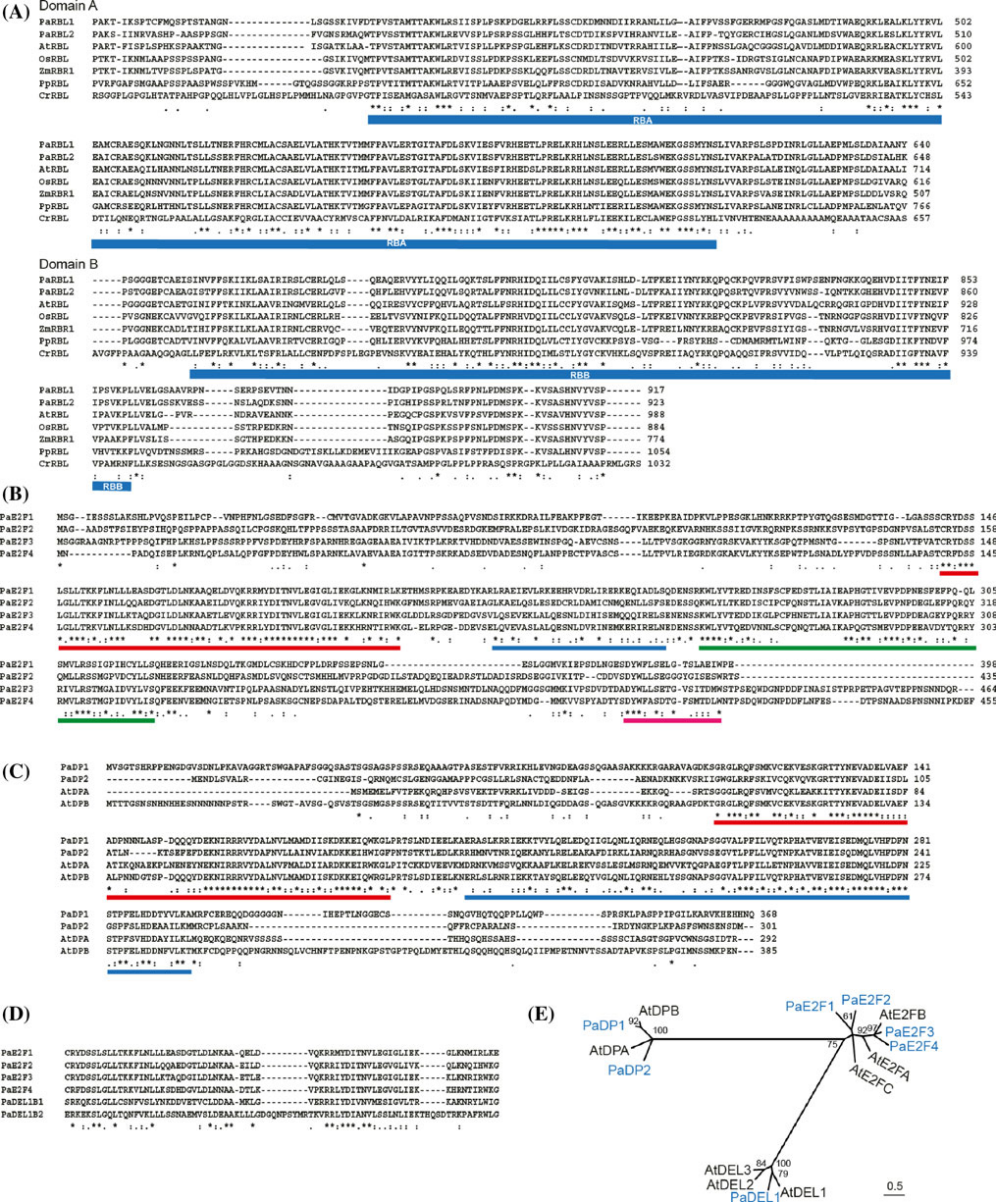
ClustalW alignment of **a** the conserved A and B domains (marked by *blue bar*) of RB proteins, **b** E2F proteins, **c** DP proteins, and **d** the conserved DNA binding domain of E2F and DEL1 proteins. *Asterisk* indicates a conserved residue. *Colon* indicates a residue showing strong similarity among sequences. *Dot* indicates a residue showing weak similarity among sequences. Pa, *P. aphrodite*; At, *A. thaliana*; Os, *O. sativa*; Zm, *Z. mays*; Pp, *P. patens*; Cr, *C. reinhardtii.* The DNA binding domain is marked by a *red bar*. The dimerization domain is marked by a *blue bar*. The marked *box* is marked by a *green bar*. The conserved C-terminal region of E2F protein important for binding to the RB protein is marked by a *pink bar*. PaDEL1-DB1, the first DNA binding domain of PaDEL1 protein. PaDEL1-DB2, the second DNA binding domain of PaDEL1 protein. **e** Unrooted maximum-likelihood tree of the E2F, DP, and DEL families. Only the conserved regions with unambiguous alignments were used for phylogenetic analysis

Two RB-related genes were identified from the *P. aphrodite* transcriptome database (Fig. 3a, Supplementary Table S2). Because the sequence of *PaRBL2* cDNA in the database was incorrect, the full-length cDNA was identified and confirmed by reverse transcription PCR followed by sequencing. Both PaRBL1 and PaRBL2 contained canonical A and B domains that showed high similarity to RB proteins of other plant species (Fig. 3a). The A and B domains are important to form a binding pocket for E2F transcription factors. Four E2F and three DP genes were identified from the *P. aphrodite* transcriptome database (Supplementary Table S2). The full-length cDNAs of *PaE2F1*, *PaE2F2*, and *PaE2F3* were isolated and the encoded proteins contained a DNA binding domain, a dimerization domain, a marked box, and a conserved C-terminal region that potentially mediates their interactions with the PaRBL proteins (Fig. 3b). The full-length cDNAs of *PaDP1* and *PaDP2* were also isolated and verified. Both *PaDPs* encode a protein containing the DP canonical DNA binding and dimerization domains (Fig. 3c). We were not able to obtain the full-length cDNAs of *PaE2F4* and *PaDP3*. Therefore, *PaE2F4* and *PaDP3* were not included in the following analyses.

In addition to the typical E2F and DP gene family members, an atypical DP-E2F-like (DEL) gene was identified in the *P. aphrodite* transcriptome database. Like other plant atypical DEL proteins (Vandepoele et al. 2002; Lammens et al. 2009), *PaDEL1* contained a duplicated DNA binding domain highly similar to that of the E2F transcription factor (Fig. 3d). Based on previous studies, the atypical DEL proteins do not heterodimerize with DP and instead form homodimers to exert their function (Di Stefano et al. 2003). In plants, DEL proteins are involved in regulation of cell wall biosynthesis and endoreduplication (Ramirez-Parra et al. 2004; Vlieghe et al. 2005; Lammens et al. 2008).

#### *WEE1* and *CKI* gene families

**Fig. 4:**
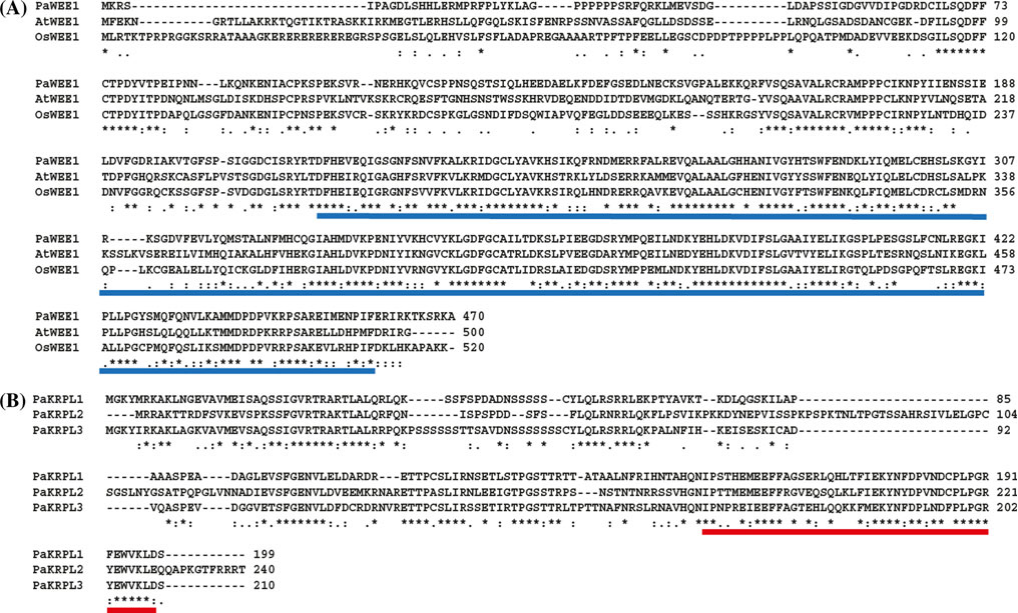
ClustalW alignment of **a** WEE1 proteins and **b** KRP proteins. *Asterisk* indicates a conserved residue. *Colon* indicates a residue showing strong similarity among sequences. *Dot* indicates a residue showing weak similarity among sequences. The catalytic domain of the WEE1 proteins is marked by a *blue bar*. The conserved CKI domain of the KRP proteins is marked by a *red bar*

One *WEE1* ortholog was found in the *Phalaenopsis* annotation database. The encoded protein displays 47 % identity to AtWEE1 and 57 % identity to rice WEE1 proteins at the amino acid sequence level. The *Phalaenopsis* WEE1 protein has a conserved catalytic domain including the ATP binding site of the protein kinase family (Fig. 4a). It also contains a conserved E/DGD triplet motif that distinguishes WEE1-related kinases from other kinase families (Booher et al. 1993; Sorrell et al. 2002).

Three potential CDK kinase inhibitor (CKI) genes were annotated and identified in *P. aphrodite*. All three carried a CKI domain (pfam02234) at the C-terminal region (Fig. 4b). PaKRP1 shares 69 % amino acid sequence identity with PaKRP3. PaKRP2 shares 52 and 49 % amino acid sequence identity with PaKRP1 and PaKRP3, respectively.

### Cell-cycle gene expression profiles

**Fig. 5:**
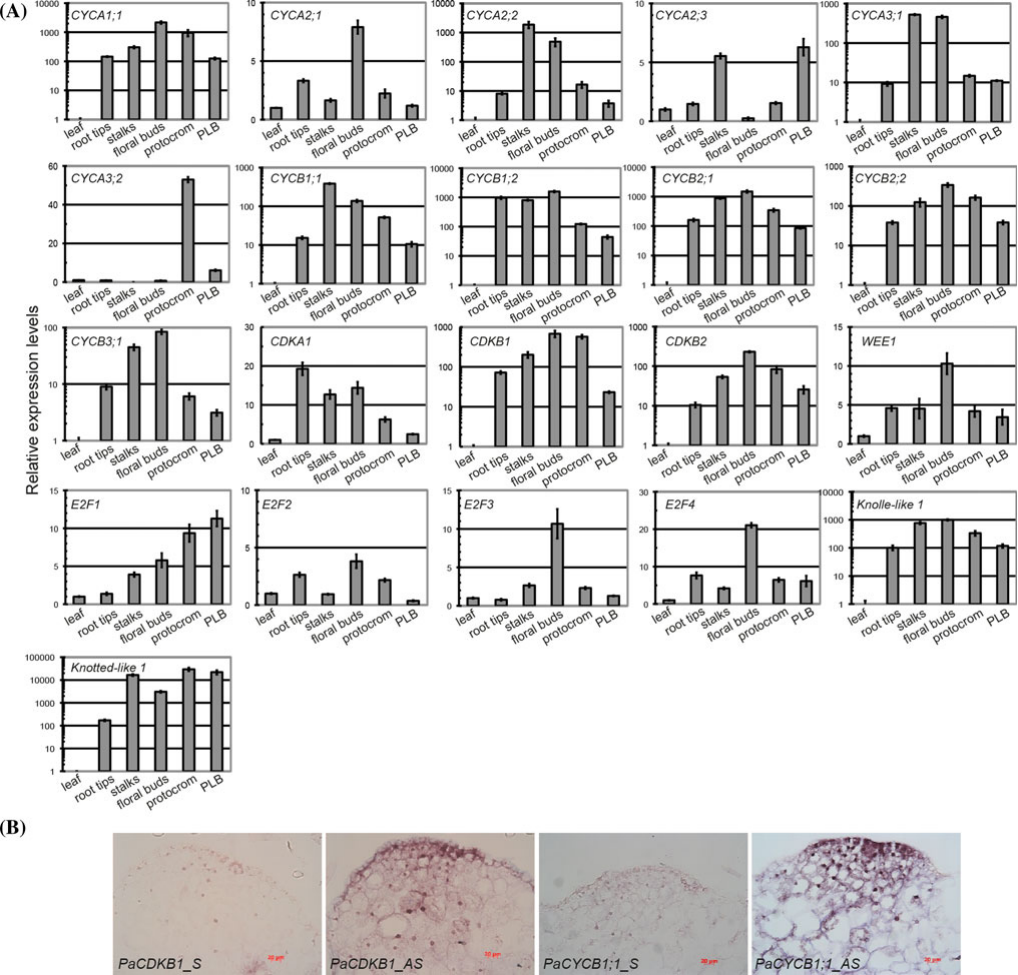
**a** Expression analysis of various cell-cycle genes in mature leaves (*leaf*), 2 cm-long root tips (*root tips*), floral stalks (*stalks*), floral buds, protocorms, and 1-month-old protocorm-like bodies (PLB) by quantitative RT-PCR. Ubiquitin (PATC150470) was used for normalization. The relative expression levels of *PaCDKA1*, *PaCYCA2;1*, *PaE2F2*, and *PaE2F3* are plotted on a liner scale. The rest of the genes are plotted on a logarithmic scale. **b** In situ hybridization with an anti-sense *PaCDKB1* or *PaCYCB1;1* probe (labeled as AS) on longitudinal sections through the center of the anterior end of 28-day-old protocorms. Sense probe of *PaCDKB1* or *PaCYCB1;1* (labeled as S) was used as a negative control

To investigate the expression patterns of the cell-cycle genes in *Phalaenopsis* orchid, we used quantitative RT-PCR (qRT-PCR) analysis to compare relative transcript abundance in various tissues. Meristematic tissues with a high rate of cell division were marked by cytokinesis-specific syntaxin (PATC147993), KNOLLE (Lauber et al. 1997), and a meristematic marker, KNOTTED-like (PATC127065) homeobox transcription factor (Fig. 5a). Our expression analysis showed that *PaCYCA1;1*, *PaCYCB1;1*, *PaCYCB1;2*, *PaCYCB2;1*, *PaCYCB2;2*, *PaCDKB1*, and *PaCDKB2* mRNAs accumulated to relatively high levels in meristems of root tips, young stalks, young floral buds, PLBs, and protocorm-containing actively dividing cells, but were almost absent or barely detectable in the fully differentiated leaves (Fig. 5a, Supplementary Fig. S2). *PaCDKB1* and *PaCYCB1;1* mRNAs were concentrated in the shoot apical meristems of the developing protocorms (Fig. 5b). Other distinct CYC gene expression patterns were also observed. For example, transcripts of *PaCYCA2;2*, *PaCYCA3;1*, and *PaCYCB3;1* were highly enriched in floral stalks and floral buds (Fig. 5a). The accumulation of *PaCYCA2;3* mRNA was enriched in floral stalks and PLBs. *PaCYCA3;2* mRNA showed preferential accumulation in developing protocorms. In addition to CYC genes, *PaE2F1*, *PaE2F2*, *PaE2F3* and *PaE2F4* also showed distinct expression patterns in the surveyed tissues (Fig. 5a). The mRNA of *PaCDKA1* was detected in both meristematic and differentiated tissues (Supplementary Fig. S2). This is consistent with expression patterns of *CDKA* genes in the other plant species (Mironov et al. 1999). However, the levels of *PaCDKA1* mRNA varied slightly in different tissues (Fig. 1).

### PaCDKA1 is a *bona fide* cyclin-dependent protein kinase

**Fig. 6:**
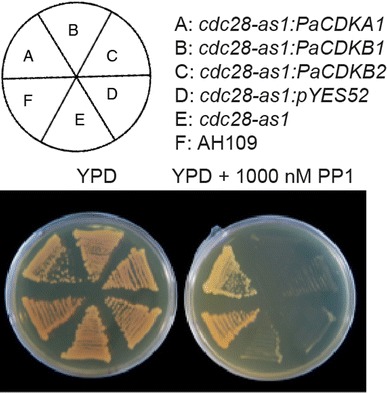
Complementation tests of PaCDKA1, PaCDKB1, and PaCDKB2 in the yeast *cdc28*-*as1* mutant

To test whether *PaCDKA1* and two *PaCDKB* genes encode functional equivalents of the evolutionarily conserved CDKs, *PaCDKA1, PaCDKB1* and *PaCDKB1* genes were expressed in an inhibitor-sensitive allele of yeast CDK, *cdc28*-*as1* mutant (Bishop et al. 2000). The *cdc28*-*as1* allele has an enlarged ATP-binding pocket, allowing it to bind the cell permeable ATP analog 1NM-PP1, and treatment of cells with 1NM-PP1 results in rapid and specific down-regulation of Cdc28 kinase activity. In the presence of 1NM-PP1, the yeast strain carrying the *cdc28*-*as1* allele failed to grow. Introduction of *PaCDKA1* rescued the proliferation defect of cdc28 mutant when 1NM-PPA was present (Fig. 6). This experiment demonstrated that *PaCDKA1* is a functional homolog of the cdc28 CDK family. However, neither *PaCDKB1* nor *PaCDKB2* rescued inhibitor-sensitive alleles of the yeast cdc28 mutant (Fig. 6). Failure to functionally complement the cdc28 mutant of budding yeast has been previously shown for CDKBs isolated from other plant species (Imajuku et al. 1992; Fobert et al. 1996). Lack of complementation in these cases indicates that plant specific B-type CDKs are functionally divergent from the A-type CDK family and cannot replace cdc28 in budding yeast.

### PaDPs interact with PaE2F1, PaE2F2, and PaE2F3, and this interaction stimulates nuclear translocation of PaE2Fs

**Fig. 7:**
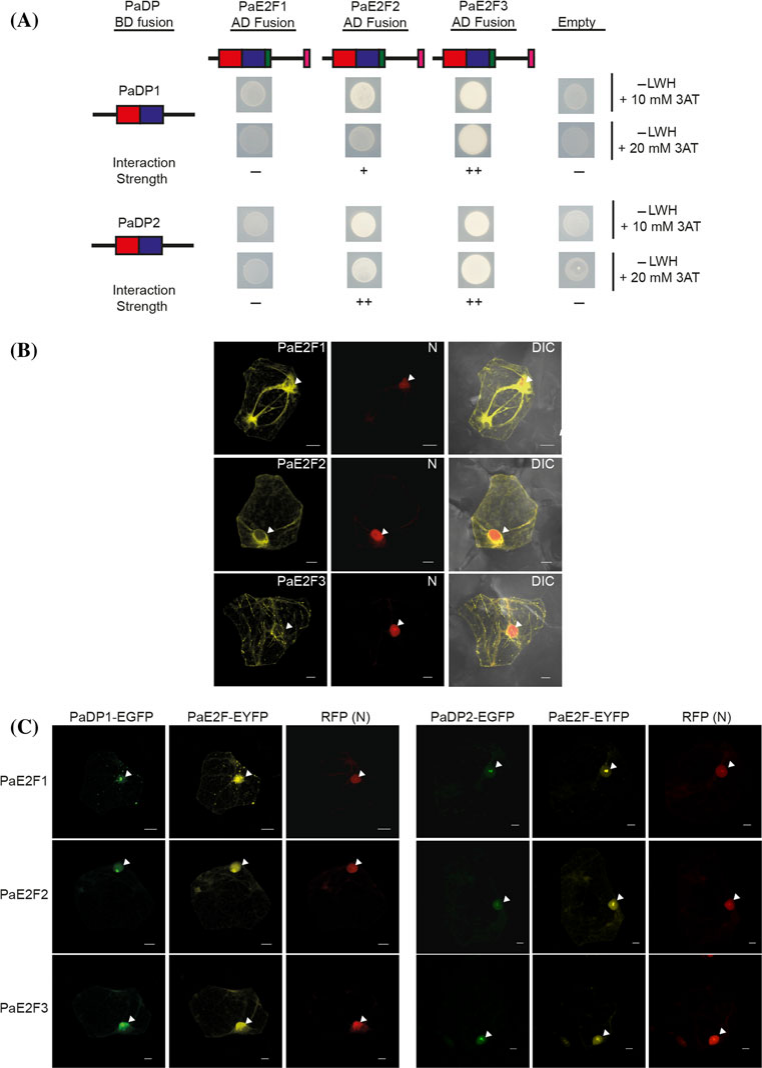
**a** Y2H interaction assay with PaE2F proteins and PaDP proteins. *Red boxes* represent the DNA binding domain and *blue boxes* represent the dimerization domain for PaE2Fs and PaDPs proteins. *Green boxes* represent the marked box and *pink boxes* at the C terminus of PaE2F proteins represent the putative PaRB binding domain (See Fig. 3). Yeast strains carrying the Gal4 activation domain (AD) and/or DNA binding domain (BD) fusion proteins were tested for growth in selective medium lacking Leu, Trp and His (−LWH), and supplemented with 3AT. The *minus* *symbol* indicates no interaction, the *plus* *symbols* indicates relative strength of interaction. **b** Subcellular localization of PaE2F1-YFP, PaE2F2-YFP and PaE2F3-FYP in *Phalaenopsis* petal cells. DIC, differential interference contrast images of cells superimposed with YFP and RFP signals. **c** Co-localization of either PaDP1-GFP or PaDP2-GFP with PaE2F1-YFP, PaE2F2-YFP or PaE2F3-YFP in *Phalaenopsis* petal cells. DIC, differential interference contrast images of cells superimposed with PaE2F (YFP), PaDP (GFP), and nuclear marker (RFP) channels. N, nucleus marked by VirD2-NLS-RFP marker. Arrowheads point out the nuclei. *Scale bar* 10 μm

*Phalaenopsis* E2F and DP have N-terminal DNA binding and dimerization domains that are evolutionarily conserved among their homologs derived from plants and animals (Fig. 3b, d). We used a Y2H assay to test the interaction between each PaE2F and each PaDP protein. PaDP1 or PaDP2 protein was fused to the yeast Gal4 DNA binding domain (DB) as a bait construct. PaE2F1, PaE2F2, or PaE2F3 protein was fused to the yeast Gal4 activation domain (AD) as a prey construct. The plasmids were then transformed into a yeast strain that has HIS3 and URA3 under the control of GAL4 binding sites as reporters. Neither co-transformation of *PaDP*-*DB* with empty AD constructs nor *PaE2F*-*AD* with empty BD constructs caused reporter activation in the Y2H assay (Fig. 7a, Supplementary Fig. S3). On the other hand, co-expression of the *PaE2F2*-*AD* or *PaE2F3*-*AD* construct with either the *PaDP1*-*DB* or *PaDP2*-*DB* construct showed weak to strong reporter activation (Fig. 7a). This indicates a specific interaction between PaDP proteins, and PaE2F2 and PaE2F3 proteins. There was no detectable reporter activation when *PaE2F1*-*AD* and *PaDP1*-*DB* or *PaE2F1*-*AD* and *PaDP2*-*DB* constructs were co-transformed (Fig. 7a). Interestingly, *PaE2F3*-*DB* bait construct exhibited auto-activation activity, but *PaDP1*-*DB* did not in the Y2H assay (Supplementary Fig. S3).

We next examined the subcellular localization of PaE2F and PaDP1 protein in *Phalaenopsis* petal tissues using bombardment-mediated transient expression assay (Hsu et al. 2011b). Enhanced yellow fluorescent protein (EYFP) was C-terminally fused to the full-length *PaE2F1*, *PaE2F2*, or *PaE2F3* cDNA. Enhanced green fluorescent protein (EGFP) was C-terminally fused to the full-length *PaDP1* or *PaDP2* cDNA. In the absence of the *PaDP*-*EGFP* construct, the subcellular localization patterns of PaE2F1-EYFP, PaE2F2-EYFP, and PaE2F3-EYFP proteins resembled those of the EYFP protein control for most of the examined cells (Fig. 7b). Occasionally, nuclear localization of PaE2F1-EYFP, PaE2F2-EYFP, and PaE2F3-EYFP proteins was observed (data not shown). The occasional nuclear localization of single PaE2F1-EYFP, PaE2F2-EYFP, or PaE2F3-EYFP protein is likely due to its interaction with the endogenous PaDP proteins. We then examined the change in the subcellular localization of PaE2F1-EYFP, PaE2F2-EYFP, and PaE2F3-EYFP by co-bombardment with the *PaDP1*-*EGFP* or *PaDP2*-*EGFP* construct. Expression of the PaDP1-EGFP or PaDP2-EGFP protein greatly facilitated the nuclear translocation of PaE2F1-EYFP, PaE2F2-EYFP, and PaE2F3-EYFP (Fig. 7c). These results indicate that PaDP1 and PaDP2 proteins can interact with each PaE2F proteins and this interaction stimulates nuclear translocation of the PaE2Fs proteins. Taken together, our data suggest that PaDP1 and PaDP2 proteins interact with PaE2F1, PaE2F2, or PaE2F3 respectively and that this interaction is crucial for nuclear translocation of the PaE2F proteins in *P. aphrodite*.

### Interaction map of the major CDKs and CYCs of *P. aphrodite*

**Fig. 8:**
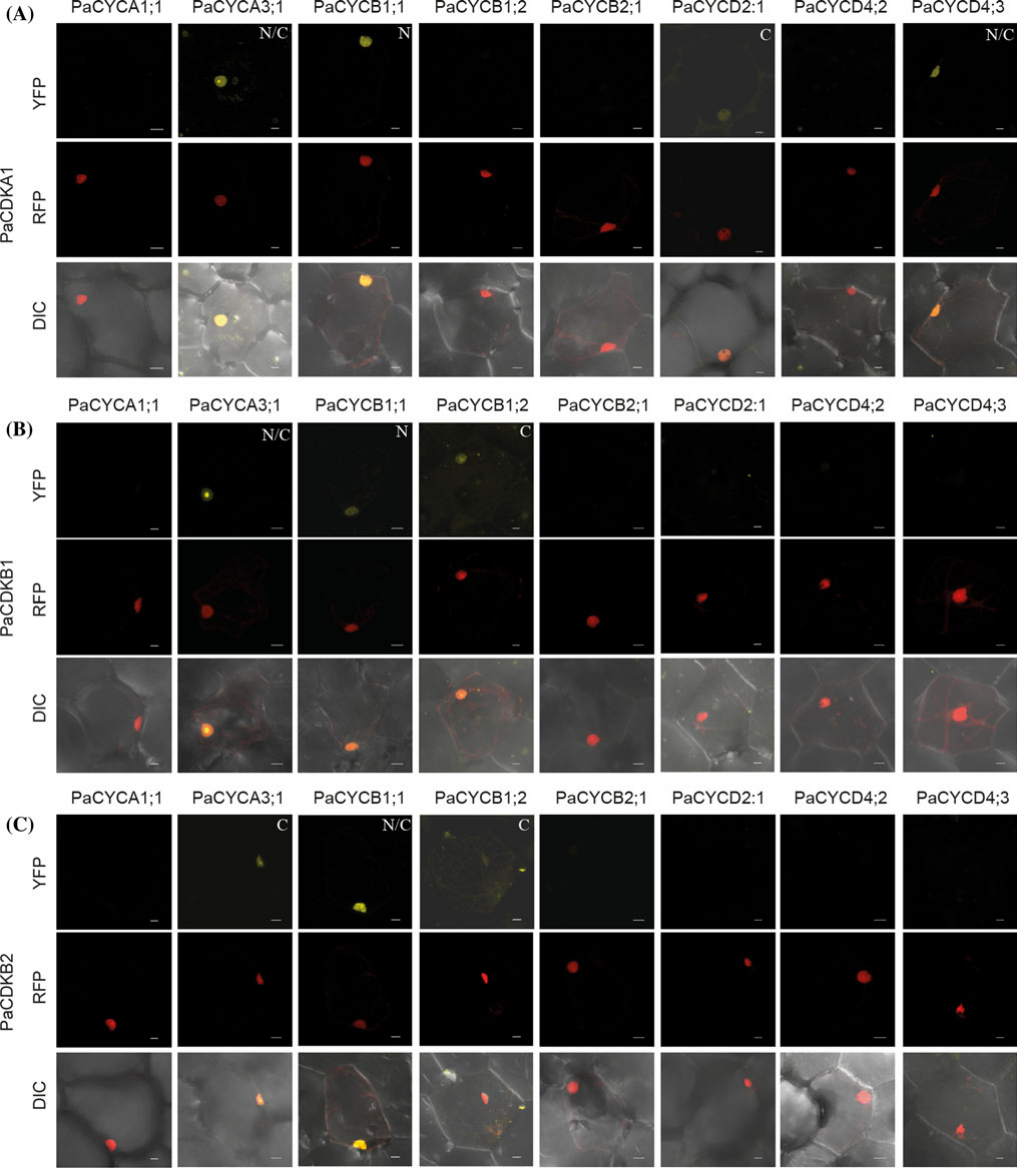
Protein–protein interactions of CDK/CYC proteins visualized using BiFC. The images of BiFC-signals are from *Phalaenopsis* petal cells co-bombarded with constructs of various combinations: **a** the *N*-*(nEYFP)*-*CDKA1* and *N*-*cEYFP*-*CYC* or *CYC*-*C*-*cEYFP* constructs, **b** the *N*-*(nEYFP)*-*CDKB1* and *N*-*cEYFP*-*CYC* or *CYC*-*C*-*cEYFP* constructs, **c** the *N*-*(nEYFP)*-*CDKB2* and *N*-*cEYFP*-*CYC* or *CYC*-*C*-*cEYFP* constructs. N, positive interacting signal from N-(nEYFP)-CDKs and N-cEYFP-CYC proteins. C, positive interacting signal from N-(nEYFP)-CDKs and CYC-C-cEYFP proteins. VirD2-NLS-RFP was used as a nuclear marker. DIC, differential interference contrast images of cells superimposed with YFP and RFP channels. *Scale bar* 10 μm

CDK activity requires interaction with CYC proteins. To uncover specific CDK/CYC protein complexes that operate during the cell-cycle transitions in *P. aphrodite*, we used BiFC assays to generate an interaction map. We only tested proteins whose full-length cDNA could be isolated. PaCDKA1, PaCDKB1, and PaCDKB2 proteins were N-terminally tagged with the N terminal half of EYFP (nEYFP; amino acids 1–174). PaCYCA1;1, PaCYCA3;1, PaCYCB1;1, PaCYCB1;2, PaCYCB2;1, PaCYCD2;1, PaCYCD4;2, and PaCYCD4;3 were N-terminally or C-terminally tagged with the C-terminal half of EYFP (cEYFP; amino acids 175–239). Only the N-terminally tagging version was obtained for *PaCYCB1;2* and *PaCYCD2;1* genes. The constructs were co-bombarded and transiently expressed in the petal epidermal cells of *Phalaenopsis*. Protein–protein interactions were visualized using confocal microscopy (Fig. 8). The construct containing the CDK gene co-bombarded with a construct containing only the C-terminal half of EYFP was used as a negative control. No fluorescence could be detected when only the C-terminal half of the EYFP was co-bombarded with CDK constructs (Supplementary Fig. S4). Positive interaction was scored by the presence of yellow florescence from co-bombardment of the CDK construct and either one of the tested CYC constructs. Of the 24 interactions tested, ten interacting pairs were identified. PaCDKA1 showed strong interaction with PaCYCA3;1, N(cEYFP)-PaCYCB1;1, and PaCYCD4;3 (Fig. 8). PaCDKA1 also showed a very weak but noticeable interaction with PaCYCD2;1 (Fig. 8). Both PaCDKB1 and PaCDKB2 were able to interact with PaCYCA3;1, PaCYCB1;1, and PaCYCB1;2: PaCDKB1 showed a weak interaction with N(cEYFP)-PaCYCB1;1 or PaCYCB1;2-C(cEYFP); and PaCDKB2 showed a weak interaction with PaCYCA3;1-C(cEYFP) or PaCYCB1;2-C(cEYFP). Interestingly, the interacting florescence signals of PaCDKB1 and CYCA3;1 were predominantly localized in a defined region of nuclei, possibly the nucleoli. All of the observed interaction signals were localized in the nuclei where CDK activity took place. Based on BiFC assays, neither PaCDKB1 nor PaCDKB2 showed clear florescence signals indicating interaction with PaCYCA1;1, PaCYCB2;1, PaCYCD2;1, PaCYCD4;2 or PaCYCD4;3.

### The core cell-cycle genes are coordinately regulated during ovule and seed development

**Fig. 9:**
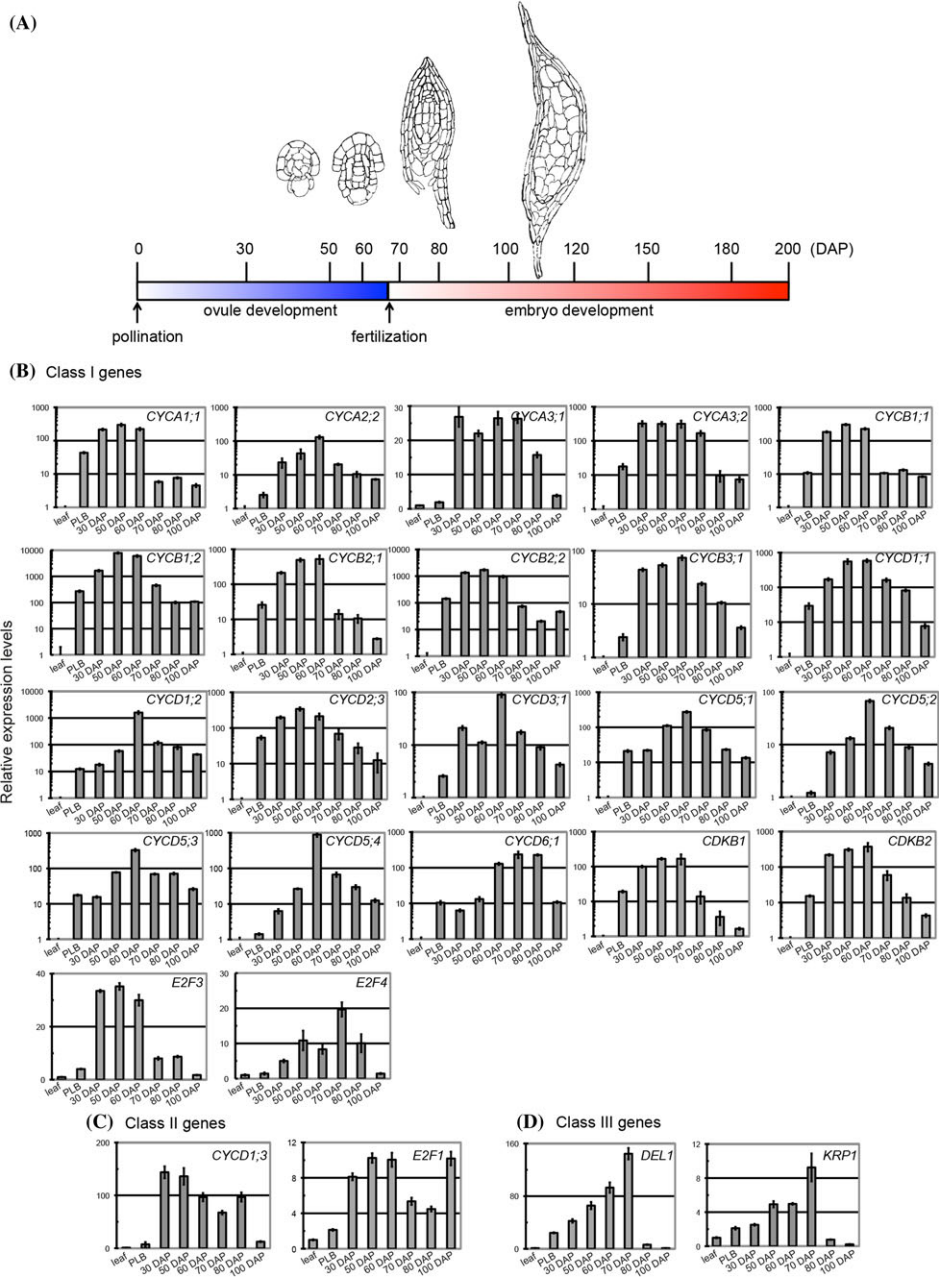

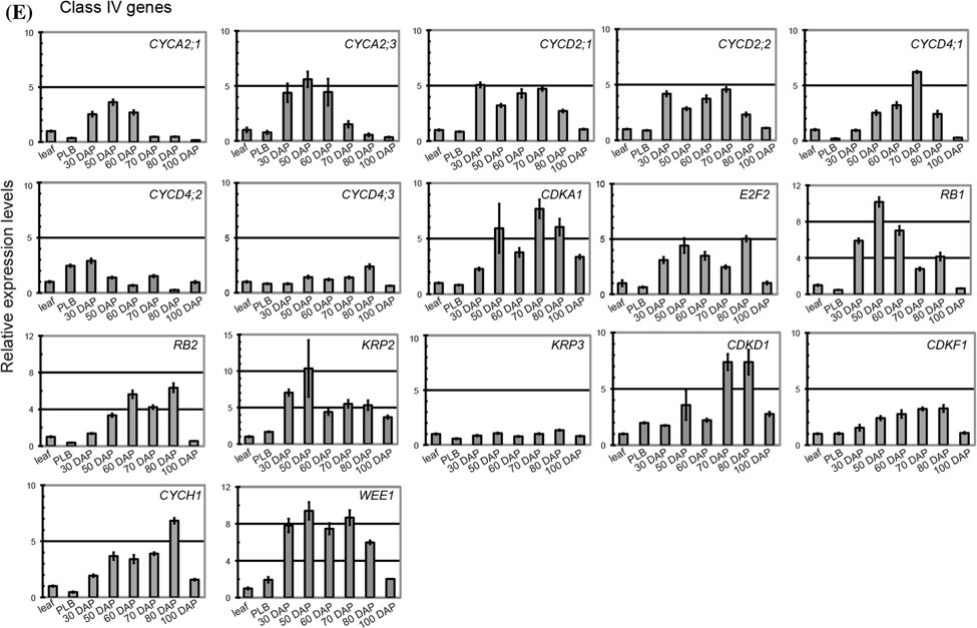
**a** Schematic diagram of the timeline of reproductive development in *Phalaenopsis* orchid. Expression analysis of **b** class I, **c** class II, **d** class III, **e** class IV cell-cycle genes in developing ovaries, mature leaves, and 1-month-old PLBs by quantitative RT-PCR. Ubiquitin (PATC150470) was used for normalization. The relative expression levels of *PaCYCA1;1*, *PaCYCA2;2*, *PaCYCA3;2*, *PaCYCB1;1*, *PaCYCB1;2*, *PaCYCB2;1*, *PaCYCB2;2*, *PaCYCB3;1*, *PaCYCD1;1*, *PaCYCD1;2*, *PaCYCD2;3*, *PaCYCD3;1*, *PaCYCD5;1*, *PaCYCD5;2*, *PaCYCD5;3*, *PaCYCD5;4*, *PaCYCD6;1*, *PaCDKB1*, and *PaCDKB2* are presented on a logarithmic scale. The relative levels of the other genes are presented on a linear scale. Data are from technical triplicates and are presented as mean normalized levels ± SEM

Cell-cycle regulators are essential for increasing cell number and establishing developmental programs during embryo development (Ebel et al. 2004; Gutzat et al. 2011; Eloy et al. 2012). *Phalaenopsis* orchids have a very unique ovule and embryonic development program: pollination-induced cell division in the placental ridge initiates ovule development, and fertilization does not occur until 60–70 days after pollination (Nadeau et al. 1996; O’Neill 1997; Lee et al. 2008). This unique developmental program suggests that the cell-cycle program is coordinated to accommodate this special developmental decision. To gain insights into how cell-cycle regulators are regulated during ovule and embryo development in *Phalaenopsis* orchids, we analyzed the transcript abundance of selected cell-cycle genes during reproductive development after pollination (Fig. 9). As a comparison, RNA samples from mature leaves were used as a baseline. Because PLBs have been referred to as somatic embryos in orchid species (Chang and Chang 1998; Ishii et al. 1998; Chen et al. 1999), one-month-old PLB samples were also included in this study. Cell-cycle regulators such as *PaCYCA1;1, PaCYCA2;2, PaCYCA3;1*, *PaCYCA3;2*, *PaCYCB1;1*, *PaCYCB1;2*, *PaCYCB2;1*, *PaCYCB2;2*, *PaCYCB3;1*, *PaCYCD1;1*, *PaCYCD1;2*, *PaCYCD2;3*, *PaCYCD3;1*, *PaCYCD5;1*, *PaCYCD5;2*, *PaCYCD5;3*, *PaCYCD5;4*, *PaCYCD6;1*, *PaCDKB1*, *PaCDKB2*, *PaE2F3,* and *PaE2F4* accumulated after pollination as ovule primordia started to develop and enlarge. Their levels reached peaks at approximately 60–70 days after pollination (DAP) when fertilization occurred and then decreased gradually or sharply as embryo development initiated. The core cell-cycle genes that shared this expression pattern were categorized as class I genes. Similar to class I cell-cycle genes, accumulation of *PaCYCD1;3* and *PaE2F1* mRNAs also reached their first peak during ovule development; however, their expression levels declined and then reached a second peak at 80 or 100 DAP. Genes that shared this expression pattern were categorized as class II cell-cycle genes. Most of the cell-cycle regulators described above were hardly detectable or expressed in low abundance in the mature leaf tissues and showed relatively low to moderate expression levels in one-month-old PLBs. The expression patterns of a third class of cell-cycle regulators including *PaDEL1* and *PaKRP1* resembled class I genes with the expression plateauing at approximately 70 DAP; however, unlike the class I genes, the expression peaks of these cell-cycle regulators were followed by a steep drop in mRNA abundance. These cell-cycle regulators were classified as class III cell-cycle genes. *PaCYCA2;1*, *PaCYCA2;3*, *PaCYCD2;1*, *PaCYCD2;2*, *PaCYCD4;1, PaCYCD4;2, PaCYCD4;3, PaCDKA1*, *PaE2F2*, *PaRB1, PaRB2,* *PaKRP2*, *PaKRP3*, *PaCDKD1*, *PaCDKF1*, *PaCYCH1*, and *PaWEE1*, on the other hand, only showed steady to slight increases (less than tenfold difference) in mRNA abundance during ovule and embryo development. These genes were classified as class IV cell-cycle genes. Among them, genes likely required for G1/S transition such as *PaCYCD2;1*, *PaCYCD2;2*, *PaCYCD4;1*, *PaE2F2*, and *PaWEE1* showed slight, but significant induction at the transcript level. Taken together, the distinct expression patterns of the cell-cycle genes define molecular aspects of cell-cycle program during gametophyte and embryo development in *Phalaenopsis* orchid.

**Fig. 10:**
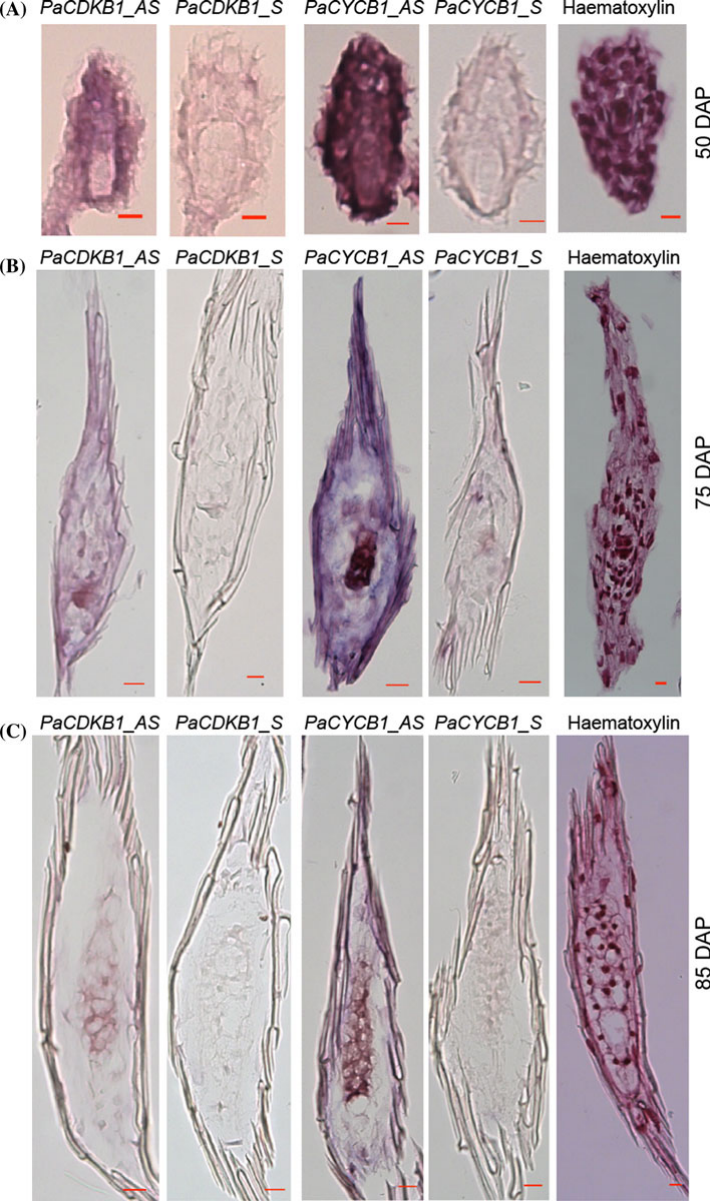
In situ hybridization with an anti-sense (labeled as AS) *PaCDKB1* or *PaCYCB1;1* probe on longitudinal sections through the center of **a** the developing female gametophytes at 50 days after pollination (DAP), **b** developing embryos at 75 DAP or **c** developing embryos at 85 DAP. Sense probes (labeled as S) of *PaCDKB1* or *PaCYCB1;1* were used as a negative control. Haematoxylin stain was used to visualize the tissue morphology. *Scale bar* 10 μm

To gain further insights into temporal and spatial gene expression patterns during mitotic cell-cycle activity in reproductive development, we monitored mitotic cell markers *PaCYCB1* and *PaCDKB1* (class I cell-cycle genes) using in situ hybridization. Fifty days after pollination, transcripts of *PaCYCB1* and *PaCDKB1* accumulated to high level in cells of mature embryo sacs and integuments (Fig. 10a). At the early stages of seed development (75 DAP), *PaCYCB1* and *PaCDKB1* mRNAs were concentrated in newly developed embryos and moderately expressed in the surrounding tissues of the developing embryos (Fig. 10b). As embryo development proceeded (85 DAP), the transcript abundance of *PaCYCB1* and *PaCDKB1* gradually diminished and was spatially restricted to the developing embryos (Fig. 10c). Taken together these results indicate that the core cell-cycle genes are temporally and spatially regulated during reproductive development in *Phalaenopsis* orchids.

## Discussion

Cell-cycle control is an integral part of plant growth and development. Despite its importance, knowledge about how cell-cycle regulation modulates growth and development, and how various developmental signals are coordinated to regulate cell-cycle programs to achieve different body plans is limited. Orchidaceae is one of the most abundant families in the plant kingdom and exhibits diverse and specialized developmental programs. Identification and analysis of orchid cell-cycle genes will provide the basis for understanding of how cell-cycle machinery is integrated into the developmental program in *Phalaenopsis* orchids. Here, we isolated and validated the cDNA sequences of some of the core cell-cycle regulators in *P. aphrodite*. Analysis of the *Phalaenopsis* core cell-cycle genes revealed that the number of the cell-cycle genes in each family category is similar to those in Arabidopsis and rice (Supplementary Table S1). Since the *Phalaenopsis* genome is not yet available, we cannot exclude the possibility that there are also other, as yet unidentified, cell-cycle genes whose expression is restricted to a defined developmental window or that are induced in response to specific environmental cues that are missing from available databases. Nevertheless, this study is, to our knowledge, the first comprehensive analysis of the cell-cycle genes in orchid species.

Similar to Arabidopsis and rice, eight types of CDKs (A-, B-, C-, D-, E-, F-, G-, and L-types) were found in *P. aphrodite*. Protein sequence analysis showed considerable conservation in the catalytic core and specific motifs of each type of CDK. A- and B-type CDKs are the master regulators that control the cell-cycle transitions in plants. One A-type CDK and two B-type CDKs were identified from the *P. aphrodite* transcriptome database. *PaCDKA1* was constitutively expressed in the examined tissues. This is consistent with observations in other plant species. *PaCDKA1* is evolutionarily conserved because it was able to functionally substitute the cdc28 protein kinase in budding yeast. *Phalaenopsis* B-type CDKs, on the other hand, could not functionally complement the yeast cdc28 mutant. Similarly, B-type CDKs from several plant species have been shown to fail to functionally replace yeast cdc28 mutant (Hirayama et al. 1991; Porceddu et al. 1999; Corellou et al. 2005). This result supports the notion that B-type CDKs are plant specific and functionally diverged from A-type CDKs (Joubes et al. 2000; Boudolf et al. 2001; Inze and De Veylder 2006). Similar to the expression patterns found in other plant B-type CDKs, *PaCDKB1 and PaCDKB2* were highly enriched in meristematic tissues with strong cell-cycle activity (Menges et al. 2002; Guo et al. 2007). It is, therefore, likely that *Phalaenopsis* B-type CDKs play a role in regulating G2/M and M phases as demonstrated in other plant species.

Five out of the nine types of CYCs found in rice and Arabidopsis (Vandepoele et al. 2002; La et al. 2006) were identified from the *P. aphrodite* transcriptome database. Failure to identify members of the four other CYC families suggests that they might be expressed at defined developmental time points or be induced under specific conditions, resulting in their lack of representation in the current transcriptome database. Among the identified CYC families, A-, B-, and D-type CYCs are the major cell-cycle regulators that play crucial roles in regulating cell-cycle transitions. Consistent with their roles during the cell cycle, most of the transcripts of *Phalaenopsis* A- and B-type CYCs were highly enriched in meristematic tissues such as floral buds, floral stalks, protocorms, and PLBs.

The transcription functions of E2F proteins require interaction with DP proteins. We confirmed that PaE2F2 and PaE2F3 proteins interacted with either PaDP1 or PaDP2 proteins in Y2H assays. Even though PaE2F1 failed to interact with either PaDP protein in Y2H analysis, co-expression of PaDP1 or PaDP2 greatly facilitated nuclear translocation of PaE2F1 in petal cells. False negative results have been reported in Y2H assays (Walhout et al. 2000), probably due to stringent scoring criteria (Boruc et al. 2010) or low sampling sensitivity (Venkatesan et al. 2009). Unlike constitutive nuclear localization of E2F-1, -2, and -3 proteins in humans (Verona et al. 1997), PaE2F proteins alone did not target exclusively into the nucleus. The nuclear translocation of PaE2F proteins requires co-expression of PaDP proteins (Fig. 7b, c). A similar subcellular localization pattern has also been reported for Arabidopsis E2F proteins (Kosugi and Ohashi 2002). Taken together these findings suggest that the nuclear localization of *Phalaenopsis* E2F proteins requires their interaction with DP proteins.

Similar to Arabidopsis, CDKA1 in *P. aphrodite* behaved as the most interconnected node in the CDK/CYC interacting network (Boruc et al. 2010; Van Leene et al. 2007, 2010, 2011). In Arabidopsis, CDKA is able to interact with D-, A-, and B-type CYCs. This shows that the functional activity of CDKA is required throughout the G1/S to mid-M phases (Porceddu et al. 2001; Sorrell et al. 2001; Boruc et al. 2010). The protein–protein interaction studies based on BiFC confirm that the functional units of CDKA protein complexes are evolutionarily conserved in *P. aphrodite*. However, PaCDKA1 seems to selectively interact with members of the D-, A-, and B-type CYCs while Arabidopsis CDKA1 is less selective for CYC binding. It is possible that insufficient flexibility and steric hindrance of the fusion proteins resulted in false negative results. It is also possible that the stable interaction of the *Phalaenopsis* CDKA/CYC pairs require interactor proteins, which are lacking in the fully differentiated petal cells. Our protein–protein interaction studies also confirmed that both PaCDKB1 and PaCDKB2 are able to interact with PaCYCB1;1, PaCYCB1;2 and PaCYCA3;1. A similar interaction network has been documented in Arabidopsis (Boruc et al. 2010; Van Leene et al. 2011). Taken together, our studies and studies from Arabidopsis suggest that the functional CDK/CYC units are evolutionarily conserved in plants.

In many orchid species, ovule development initiates after pollination occurs (Nadeau et al. 1996). It has been suggested that ovule development, redirection of pollen tube growth, and subsequent fertilization require timely coordination of hormone regulation. Because ovule initiation and subsequent fertilization are nearly synchronous and thousands of ovules are present in each ovary (Nadeau et al. 1996), orchids provide an excellent system through which to study the timely regulation of gene expression during ovule and embryo development. In this study, we monitored and categorized the expression patterns of the cell-cycle genes in developing capsules into four categories. A subset of the cell-cycle regulators controlling G1/S and S/M transitions were grouped into the class I genes. They are: *PaCYCA1;1,* *PaCYCA2;2*, *PaCYCA3;1*, *PaCYCA3;2*, *PaCYCB1;1*, *PaCYCB1;2*, *PaCYCB2;1*, *PaCYCB2;2, PaCYCB3;1*, *PaCYCD1;1*, *PaCYCD1;2*, *PaCYCD2;3*, *PaCYCD3;1*, *PaCYCD5;1*, *PaCYCD5;2*, *PaCYCD5;3*, *PaCYCA5;4*, *PaCYCD6;1*, *PaCDKB1*, *PaCDKB2*, *PaE2F3*, and *PaE2F4*. The transcript levels of the class I cell-cycle regulators were dramatically enriched during ovule development (30–60 DAP) and gradually declined as embryos started to develop (70–100 DAP). This expression pattern suggests that cell-cycle-dependent activity regulated by these genes is important for development of the female gametophytes. The biological implications of the decline in transcripts of class I cell-cycle regulators as development switches from ovule development to embryogenesis are not clear. One possible reason is that accumulation of the class I cell-cycle proteins during ovule development is sufficient to initiate embryogenesis. The other possibility is that the cellular requirement of the cell-cycle transcripts is gradually restricted to defined domains and specific time intervals. The spatial and temporal expression patterns of cell-cycle genes during embryo development have also been documented in Arabidopsis (Collins et al. 2012; Belmonte et al. 2013).

Mirroring class I genes, class II genes were up-regulated during ovule development and down-regulated after fertilization. Intriguingly, however, the mRNA of the class II cell-cycle regulators (*PaE2F1* and *PaCYCD1;3*) reached a second peak at 80–100 DAP as embryos entered the maturation stage. The molecular basis of this expression pattern is not clear. Class III cell-cycle genes (*PaDEL1* and *PaKRP1*) showed a distinct expression pattern with accumulation of transcripts reaching a peak at 70 DAP (Fig. 9d) during which ovule development ceased and fertilization occurred, followed by a steep drop after onset of embryogenesis. In the future, it will be interesting to examine whether the class II and/or the class III cell-cycle regulators are involved in developmental processes that help to define the fate transition between gametophyte and embryo development.

In summary, we have identified and isolated the core cell-cycle genes in *Phalaenopsis* orchid, and conducted a comprehensive study of their dynamic expression patterns during reproductive development. Protein sequence analysis showed that *P. aphrodite* cell-cycle regulators are highly similar to their respective orthologs in other plant species. We confirmed the functional units of the CDK/CYC and E2F/DP by Y2H and BiFC analysis. Expression patterns and subcellular localization studies indicate that the cell-cycle regulators of *P. aphrodite* are involved in cell proliferation as well as cell-cycle related developmental processes. We observed that the expression patterns of the cell-cycle genes are coordinately regulated as the reproductive system proceeds from ovule development to embryogenesis in *P. aphrodite*. These results establish the first molecular signatures of the cell-cycle program during pollination-induced reproductive development in *Phalaenopsis* orchid. Further studies of the molecular functions of the core cell-cycle genes during seed development will be important to provide clues about how the cell-cycle program is regulated and incorporated into developmental decisions.

## Electronic supplementary material

Below is the link to the electronic supplementary material.

Supplementary material 1 (TIFF 355 kb)

Supplementary material 2 (TIFF 354 kb)

Supplementary material 3 (TIFF 239 kb)

Supplementary material 4 (TIFF 585 kb)

Supplementary material 5 (DOCX 77 kb)
