# Supplementary figures and images for: Genome-wide annotation, expression profiling, and protein interaction studies of the core cell-cycle genes in Phalaenopsis aphrodite
Source: Plant Mol Biol. 2013 Sep 25;84(1):203–26. doi: 10.1007/s11103-013-0128-y (PMC3840290; doi:10.1007/s11103-013-0128-y)

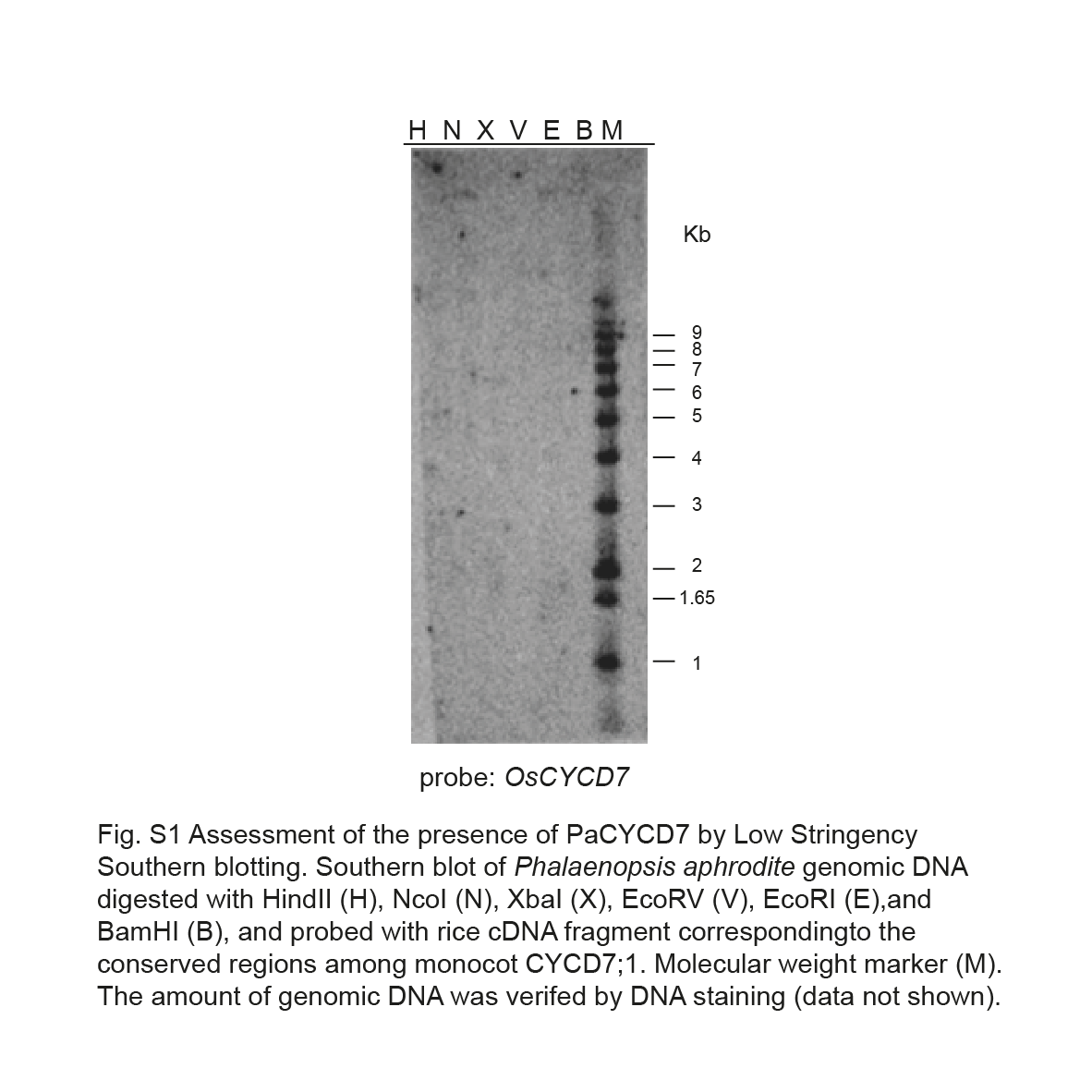

Supplement: Supplementary file 1 — Supplementary material 1 (TIFF 355 kb) [file 11103_2013_128_MOESM1_ESM.tif]

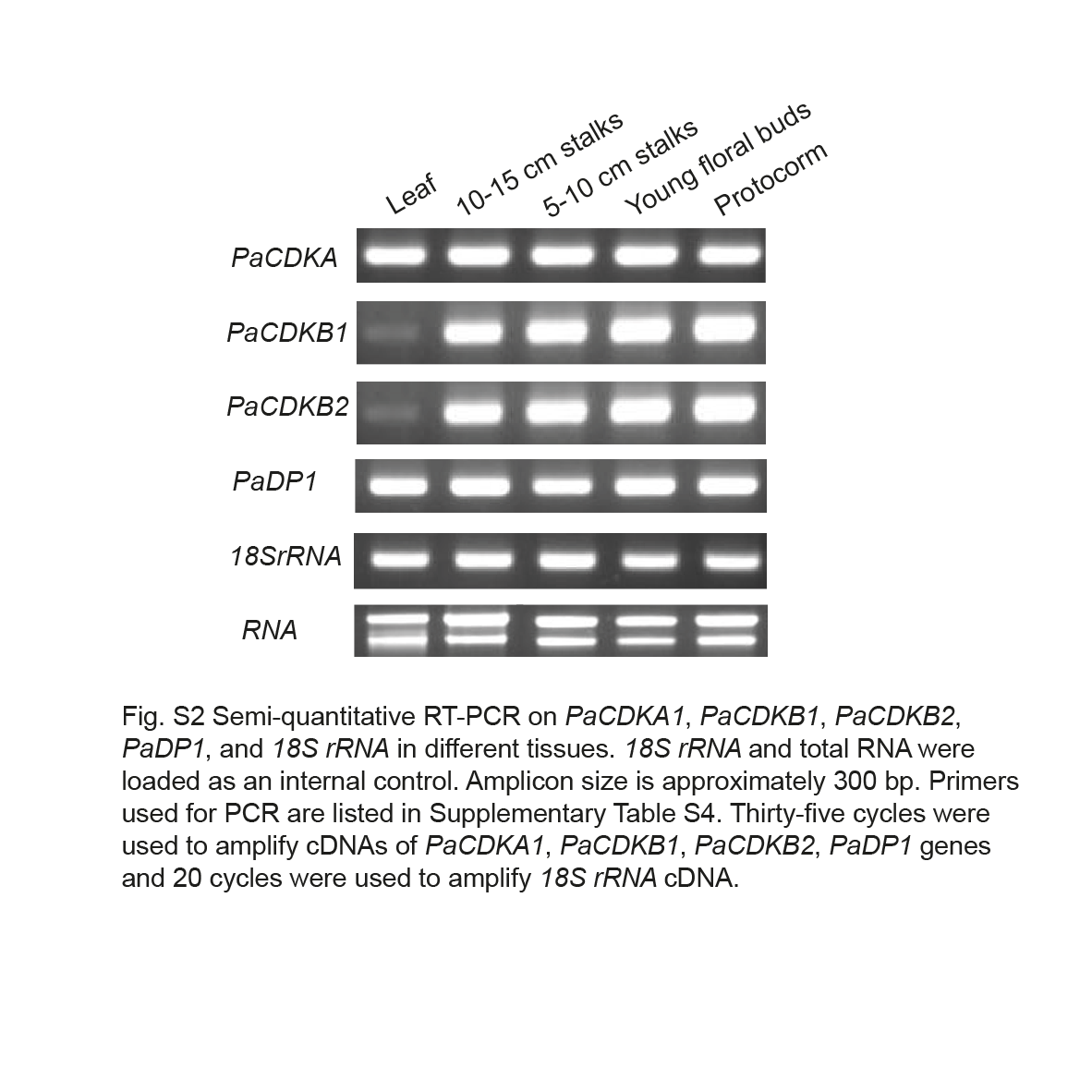

Supplement: Supplementary file 2 — Supplementary material 2 (TIFF 354 kb) [file 11103_2013_128_MOESM2_ESM.tif]

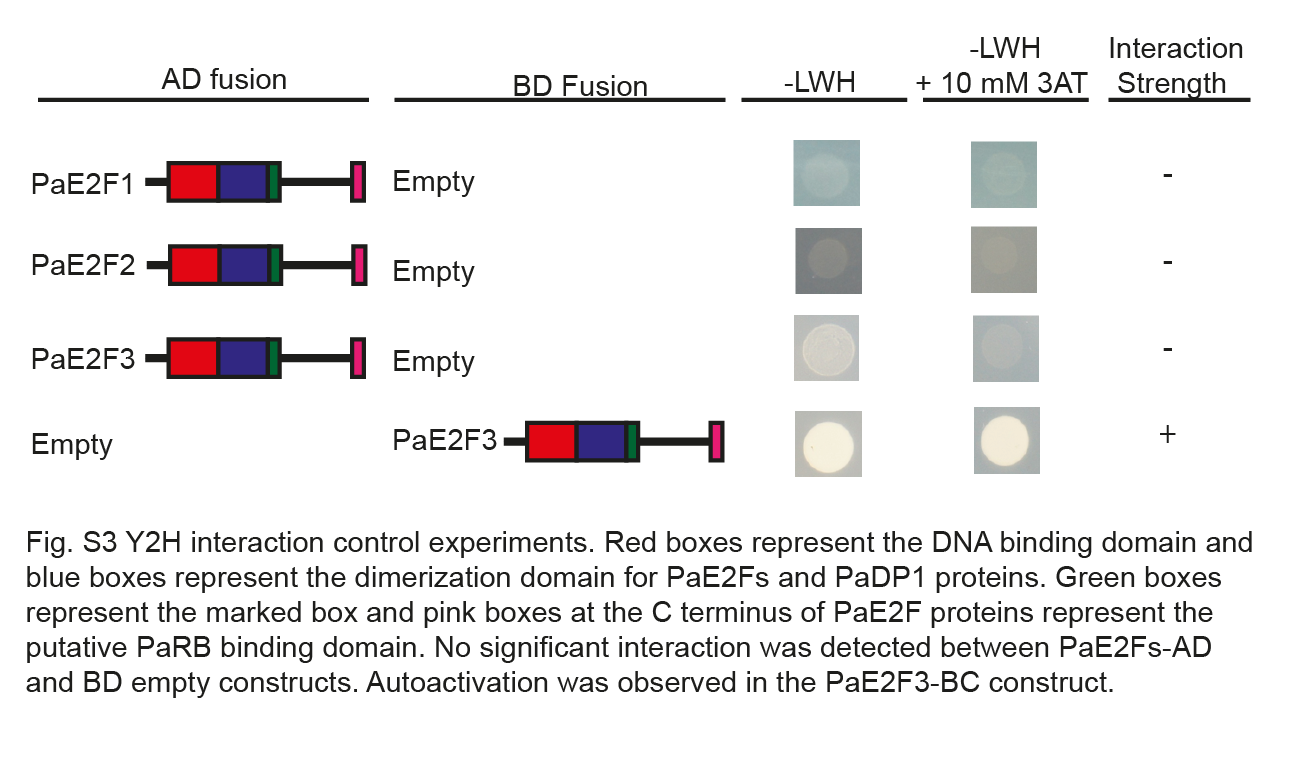

Supplement: Supplementary file 3 — Supplementary material 3 (TIFF 239 kb) [file 11103_2013_128_MOESM3_ESM.tif]

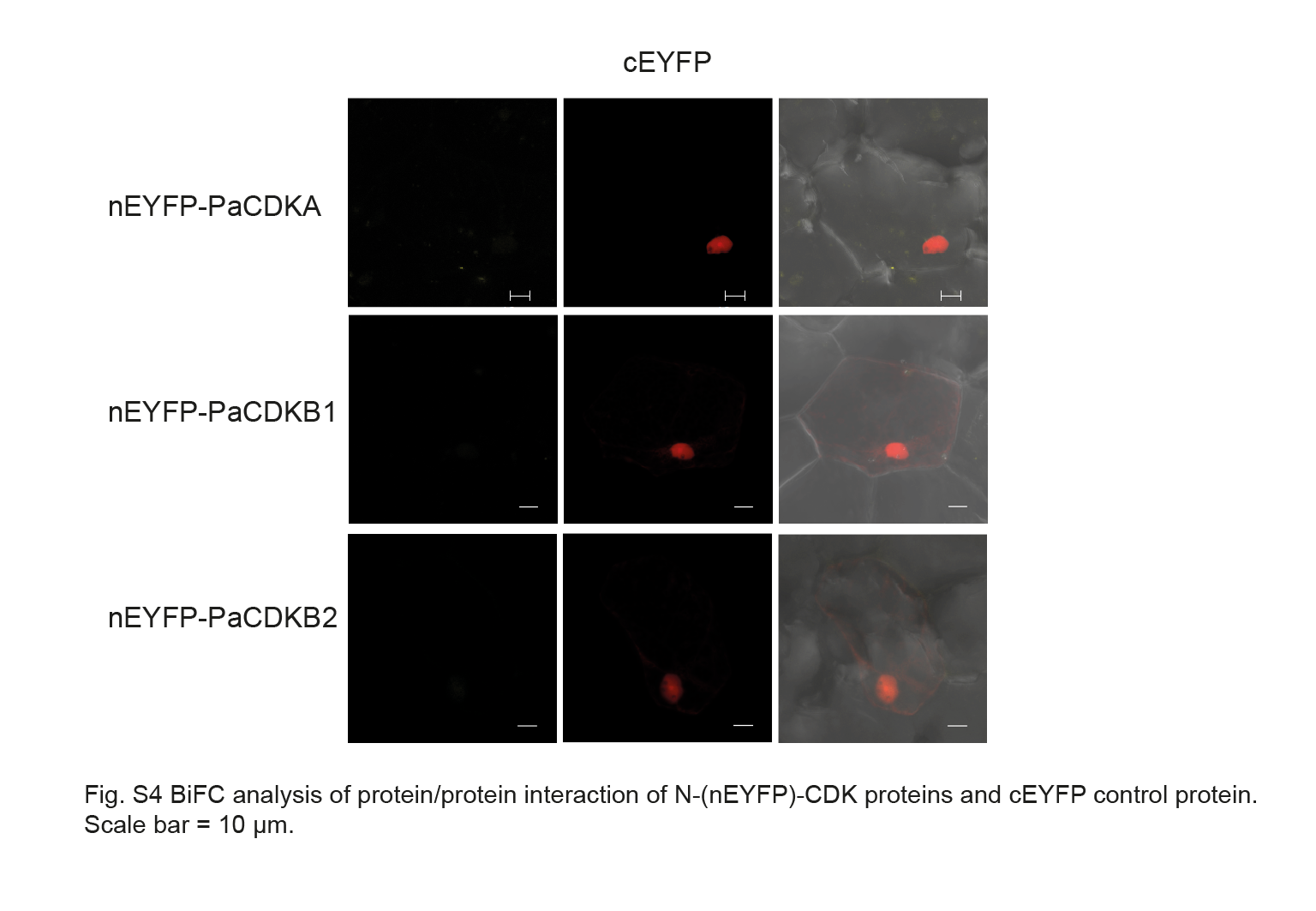

Supplement: Supplementary file 4 — Supplementary material 4 (TIFF 585 kb) [file 11103_2013_128_MOESM4_ESM.tif]
