# Supplementary material for: Genome-wide annotation, expression profiling, and protein interaction studies of the core cell-cycle genes in Phalaenopsis aphrodite
Source: Plant Mol Biol. 2013 Sep 25;84(1):203–26. doi: 10.1007/s11103-013-0128-y (PMC3840290; doi:10.1007/s11103-013-0128-y)
Supplement: Supplementary file 5 — Supplementary material 5 (DOCX 77 kb) [file 11103_2013_128_MOESM5_ESM.docx]

Supplementary Table S1. Number of cell cycle genes in Arabidopsis, Rice, and *P.* *aphrodite*

|  | Arabidopsis | Rice | *P. aphrodite*  (current status) |
| --- | --- | --- | --- |
| CDKA | 1 | 2 | 1 |
| CDKB | 4 | 2 | 2 |
| CDKC | 2 | 3 | 1 |
| CDKD | 3 | 1 | 1 |
| CDKE | 1 | 1 | 1 |
| CDKF | 1 | 4 | 1 |
| CDKG | 2 | 2 | 2 |
| CKL | 15 | 10 | 13 |
| CYCA | 10 | 7 | 6 |
| CYCB | 11 | 6 | 5 |
| CYCD | 10 | 14 | 15 |
| CYCH | 1 | 1 | 1 |
| CYCL | 1 | 2 | 1 |
| RB | 1 | 2 | 2 |
| E2F | 3 | 4 | 4 |
| DP | 2 | 3 | 3 |
| DEL | 3 | 2 | 1 |
| CKS | 2 | 1 | 1 |
| CKI | 7 | 7 | 3 |
| Wee1 | 1 | 1 | 1 |

Supplementary Table S2. List of core cell cycle genes identified from the Orchidstra *Phalaenopsis* Genome Annotation Database

|  | Gene name | Protein family | Signature motif | Putative functions | FL cDNAs isolation |
| --- | --- | --- | --- | --- | --- |
| PATC137906 | CDKA1 | CDK | PSTAIRE | G1/S, G2/M | Y |
| PATC131752 | CDKB1 | CDK | PPTTLRE | G2/M | Y |
| PATC140676 | CDKB2 | CDK | PATTLRE | G2/M | Y |
| PATC124460 | CDKC1 | CDK | PITAIRE |  |  |
| PATC127318 | CDKD1 | CDK | NFTALRE | CAK |  |
| PATC125443 | CDKE1 | CDK |  |  |  |
| PATC129117 | CDKF1 | CDK |  | CAK |  |
| PATC141662 | CDKG1 | CDK |  |  |  |
| PATC153427 | CDKG2 | CDK |  |  |  |
| PATC134163 | CYCA1;1 | Cyclin | LVEVAEEY | S/G2/M | Y |
| PATC148728 | CYCA2;1 | Cyclin | LVEVAEEY | S/G2/M |  |
| PATC152012 | CYCA2;2 | Cyclin | LVEVAEEY | S/G2/M |  |
| PATC131436 | CYCA2;3 | Cyclin | LVEVAEEY | S/G2/M |  |
| PATC024875 | CYCA3;1 | Cyclin | LVEVAEEY | S/G2/M | Y |
| PATC076443 | CYCA3;2 | Cyclin | LVEVAEEY | S/G2/M |  |
| PATC146999 | CYCB1;1 | Cyclin | HXKF | G2/M | Y |
| PATC165225 | CYCB1;2 | Cyclin | HXKF | G2/M |  |
| PATC132253 | CYCB2;1 | Cyclin | HXKF | G2/M | Y |
| PATC145167 | CYCB2;2 | Cyclin | HXKF | G2/M |  |
| PATC185698/PATC228763 | CYCB3;1 | Cyclin |  | G2/M |  |
| PATC151104 | CYCD1;1 | Cyclin | LXCXE | G1/S |  |
| PATC115849/PATC131872 | CYCD1;2 | Cyclin | LXCXE | G1/S |  |
| PATC129324 | CYCD1;3 | Cyclin | LXCXE | G1/S |  |
| PATC151467 | CYCD2;1 | Cyclin | LXCXE | G1/S | Y |
| PATC136193 | CYCD2;2 | Cyclin | LXCXE | G1/S |  |
| PATC152785 | CYCD2;3 | Cyclin | LXCXE | G1/S |  |
| PATC127489 | CYCD3;1 | Cyclin | LXCXE | G1/S |  |
| PATC155932 | CYCD4;1 | Cyclin | LXCXE | G1/S |  |
| PATC133091 | CYCD4;2 | Cyclin | LXCXE | G1/S | Y |
| PATC144249 | CYCD4;3 | Cyclin | LXCXE | G1/S | Y |
| PATC069486 | CYCD5;1 | Cyclin | LXCXE | G1/S |  |
| PATC134780 | CYCD5;2 | Cyclin | LXCXE | G1/S |  |
| PATC134512 | CYCD5;3 | Cyclin | LXCXE | G1/S |  |
| PATC006791 | CYCD5;4 | Cyclin | LXCXE | G1/S |  |
| PATC147500 | CYCD6;1 | Cyclin | LXCXE | G1/S |  |
| PATC130408 | CYCH1 | Cyclin |  |  |  |
| PATC149557 | CYCL1 | Cyclin |  |  |  |
| PATC138452 | E2F1 | E2F |  |  | Y |
| PATC132061 | E2F2 | E2F |  |  | Y |
| PATC148881 | E2F3 | E2F |  |  | Y |
| PATC049993/PATC207748 | E2F4 | E2F |  |  |  |
| PATC124220 | DP1 | DP |  |  | Y |
| PATC132383 | DP2 | DP |  |  | Y |
| PATC143537 | DP3 | DP |  |  |  |
| PATC144596 | DEL1 | E2F |  |  |  |
| PATC147585 | RbL1 | Rb |  |  |  |
| PATC125028 | RbL2 | Rb |  |  |  |
| PATC129107 | KRPL1 | KRP |  |  |  |
| PARC140694 | KRPL2 | KRP |  |  |  |
| PATC138517 | KRPL3 | KRP |  |  |  |
| PATC144561 | WEE1 | WEE1 |  | DNA damage |  |

Supplementary Table S3. Identification of genes used for phylogenic tree construction

| Synonym | Gene ID | Synonym | Gene ID |
| --- | --- | --- | --- |
| AtCDKA | PATC137906 | AtCYCD1;1 | AT1G70210.1 |
| OsCDKA | Os03g02680 | AtCYCD2;1 | AT2G22490.1 |
| ZmCDKA | NP_001105342 | AtCYCD3;1 | AT4G34160.1 |
| BcCDKA | XP_003558962 | AtCYCD3;2 | AT5G67260.1 |
| PpCDKA | XP_001780521 | AtCYCD3;3 | AT3G50070.1 |
| CrCDKA | XP_001698637 | AtCYCD4;1 | AT5G65420.1 |
| VcCDKA | XP_002949867 | AtCYCD4;2 | AT5G10040.1 |
| Cdc28 | CAA85119 | AtCYCD5;1 | AT4G37630.1 |
| HsCDK1 | NP_001777 | AtCYCD6;1 | AT4G03270.1 |
| AtCDKB1;1 | AT3G54180.1 | AtCYCD7;1 | AT5G02110.1 |
| AtCDKB1;2 | AT2G38620.1 | AtCYCA1;1 | AT1G44110.1 |
| AtCDKB2;1 | AT1G76540.1 | AtCYCA1;2 | AT1G77390.1 |
| AtCDKB2;2 | AT1G20930.1 | AtCYCA2:1 | AT5G25380.1 |
| OsCDKB1;1 | Os01g67160 | AtCYCA2;2 | AT5G11300.1 |
| OsCDKB2;1 | Os08g40170 | AtCYCA2;3 | AT1G15570.1 |
| BdCDKB1;1 | XP_003579011.1 | AtCYCA2;4 | AT1G80370.1 |
| BdCDKB1;2 | XP_003577858.1 | AtCYCA3:1 | AT5G43080.1 |
| BdCDKB2;1 | XP_003574763.1 | AtCYCA3:2 | AT1G47210.2 |
| ZmCDKB1 | NP_001141521.1 | AtCYCA3:3 | AT1G47220.1 |
| ZmCDKB2 | NP_001145781.1 | AtCYCA3:4 | AT1G47230.1 |
| SlCDKB1 | NP_001233899.1 | AtCYCB1;1 | AT4G37490.1 |
| SlCDKB2 | NP_001233905.1 | AtCYCB1;2 | AT5G06150.1 |
| AtE2FA | AT2G36010.1 | AtCYCB1;3 | AT3G11520.1 |
| AtE2FB | AT5G22220.2 | AtCYCB1;4 | AT2G26760.1 |
| AtE2FC | AT1G47870.1 | AtCYCB1;5 | AT1G34460.1 |
| AtDPA | AT5G02470.1 | AtCYCB2;1 | AT2G17620.1 |
| AtDPB | AT5G03415.1 | AtCYCB2;2 | AT4G35620.1 |
| AtDEL1 | AT3G48160.1 | AtCYCB2;3 | AT1G20610.1 |
| AtDEL2 | AT5G14960.1 | AtCYCB2;4 | AT1G76310.1 |
| AtDEL3 | AT3G01330.1 | AtCYCB2;5 | AT1G20590.1 |
| AtRBR1 | AT3G12280.1 | AtCYCB3;1 | AT1G16330.1 |
| AtWEE1 | AT1G02970.1 |  |  |
| OsWEE1 | Os02g04240 |  |  |

Supplementary Table S4. Primer pairs for qPCR or semi-qPCR

| **Gene name** | **Forward primer** | **Reverse primer** | **Amplicon size (bp)** |
| --- | --- | --- | --- |
| *PaCDKA1* | 5′-GTGGATGTGTGGTCAGTTGG-3′ | 5′-AGGCAAGGATGAAACACCTG-3′ | 153 |
| *PaCDKB1* | 5′-TTTTTGCCGAAATGGTTAGG-3′ | 5′-GGTTCCAATAAGGGGAGAGC-3′ | 193 |
| *PaCDKB2* | 5′-TGGGGCTAAAACCTATGCAG-3′ | 5′-AAATGTGGAGGAGCTGCTGT-3′ | 122 |
| *PaCDKD1* | 5′-GCACCGACTAAACCTGCTCT-3′ | 5′-GCCAATTCTAAACCGACAGG-3′ | 158 |
| *PaCDKF1* | 5′-TCAGTGTTTTGGGGGACTTC-3′ | 5′-AGGCATCCTTCCAAGCCTAT-3′ | 115 |
| *PaCYCA1;1* | 5′-GCCGAACTATCACTCCCAGA-3′ | 5′-ATGCGTAAGAGTTGCGTTCC-3′ | 129 |
| *PaCYCA2;1* | 5’-AGCGCTCCCTCTCTTTCTTT-3’ | 5’-GATTGGTCCAACGTCCATCT-3’ | 140 |
| *PaCYCA2;2* | 5′-TTCTTGCTGGATGGACATTG-3′ | 5′-TCACGTATGGCAGTGAGGAC-3′ | 157 |
| *PaCYCA2;3* | 5′-ATGGGACCATGCAAAACAATA-3′ | 5′-GTAGAGGCTGCACATTTGAGG-3′ | 140 |
| *PaCYCA3;1* | 5′-GGGAAATCCTACTGCCAAAAC-3′ | 5′-ATGCACATTTGGATTCATGGT-3′ | 193 |
| *PaCYCA3;2* | 5′-CATGCTCATTGCCTCAAAGT-3′ | 5′-GCCTTCAAGAACCTCCTCAG-3′ | 183 |
| *PaCYCB1;1* | 5′-TCGTAGCAAGGTTGCTTGTG-3′ | 5′-ATGGCTCTCATCTTCGCATT-3′ | 177 |
| *PaCYCB1;2* | 5′-GGATCGAGCTTATGCCAGAG-3′ | 5′-CCAGCTCCGCATAGAAGAAC-3′ | 170 |
| *PaCYCB2;1* | 5′-TGGTGGATTTTCACAAGCAG-3′ | 5’-CGGACAGCATTGAATCAAGA-3′ | 125 |
| *PaCYCB2;2* | 5′-TTAGCCCATCAAAGCATTCC-3′ | 5′-TAAGCTCGGTCGGAGATGAC-3′ | 131 |
| *PaCYCB3;1* | 5′-TGTTTGGTCTTACCACACTTCTTC-3′ | 5′-CATTTAACGAAGTTCAGCTTTTCA-3′ | 155 |
| *PaCYCD1;1* | 5′-TTTGCCAGCAAGGTAGATCC-3′ | 5′-GAAGGACAATGGTCCAGGAA-3′ | 113 |
| *PaCYCD1;2* | 5′-TAACCCCGAGTTTGTCTTCG-3′ | 5′-CCGTCGTCGGATAAAATGAT-3′ | 144 |
| *PaCYCD1;3* | 5′-GCCAGCTGATGGACCAGTAT-3′ | 5′-GGTGAAGGACTTCTGGGTGT-3′ | 140 |
| *PaCYCD2;1* | 5′-GATTCCTCCGTCGATTTCCT-3′ | 5′-CAGCTTCCTCCTCTTTGCTG-3′ | 145 |
| *PaCYCD2;2* | 5′-ACTCGCTAGCAGCAAAGAGG-3′ | 5′-TCTCTCCCACCAACCAAAAC-3′ | 115 |
| *PaCYCD2;3* | 5′-AGTTCAGGCCTTCAGAGGTG-3′ | 5′-GTTTTGTCCCTTGCAATGCT-3′ | 150 |
| *PaCYCD3;1* | 5′-AGCGTTCAACATGGGTTTTC-3′ | 5′-CCATCCATGGCTTGTCTTCT-3′ | 118 |
| *PaCYCD4;1* | 5′-GGATGAGGAGAGGGTGTTGA-3′ | 5′-ACTTGATTGGGAGCCAGATG-3′ | 181 |
| *PaCYCD4;2* | 5′-AGCGATGCAGGAGGAGATAA-3′ | 5′-TTTCTCTGCTGTGGGGAAGT-3′ | 187 |
| *PaCYCD4;3* | 5′-CAATCGGTGGATCTCATCCT-3′ | 5’-CGCTTTCTCAAGATCCAAGC-3′ | 129 |
| *PaCYCD5;1* | 5′-AGCCTTCTTTTGACTCCTTGC-3′ | 5′-GCGAAGCCATTTGTTGTTAAG-3′ | 122 |
| *PaCYCD5;2* | 5′-CTTACTTGGACCGGTTTCTCC-3′ | 5′-ATAAAAGCTTCATCGCCCATT-3′ | 69 |
| *PaCYCD5;3* | 5′-GAGCTCAGTGACTCCACTTCC-3′ | 5′-GAAGCTCGAAATTCCACCAA-3′ | 144 |
| *PaCYCD5;4* | 5′-TGGACACACTGGAATGGAAA-3′ | 5′-CTGAAGGGCGGTAAAACATC-3′ | 166 |
| *PaCYCD6;1* | 5′-CCAGCACTAGCCTACCTTGC-3′ | 5′-ATCGAAGATGAACCCCTCCT-3′ | 183 |
| *PaCYCH* | 5′-CCATTCCCACAGCAAAAGAT-3′ | 5′-ATCCTTCAAAAGGCGTTCCT-3′ | 156 |
| *PaE2F1* | 5′-CTCCATTGGTCCCATTCATT-3′ | 5′-CAATCATGCTTGGAGCACAA-3′ | 111 |
| *PaE2F2* | 5′-CTCCATGGGTCCTGTTGATT-3′ | 5′-GGTGCATACTCGTGCAGCTA-3′ | 125 |
| *PaE2F3* | 5′-GATTTTATGGGTGGCAGTGG-3′ | 5′-CATTGCTCAGATGGTGTGCT-3′ | 128 |
| *PaE2F4* | 5′-CTGCCATGCTTTCAGAATCA-3′ | 5′-CTATTGGACCCATGGTGCTT-3′ | 142 |
| *PaDEL1* | 5′-TTCGGCCGTCTTATCATAGC-3′ | 5′-CAGATTTCTTCAGGGGTTGC-3′ | 123 |
| *PaRB1* | 5′-CTATTCTTGCGAGGCGATTC-3′ | 5′-TCCATTAGTGCAGGTCACCA-3′ | 156 |
| *PaRB2* | 5′-GACATGTCGCCGAAGAAAGT-3′ | 5′-TTAGGCGGCTGTTAATGACC-3′ | 178 |
| *PaWEE1* | 5′-TTCTCTTGGAGCAGCCATTT-3′ | 5′-CCATCATAGCCTTCAGCACA-3′ | 146 |
| *PaKRP1* | 5′-AGGGAAACAACACCTTGCAG-3′ | 5′-TGCTGGGTATGTTTTGATGC-3′ | 133 |
| *PaKRP2* | 5′-AGTTCAGCTCATCGGAGCAT-3′ | 5′-TCGCGTTCCTTTTCATTTCT-3′ | 160 |
| *PaKRP3* | 5′-GGACTTCGATTGTCGGGATA-3′ | 5′-AAAGGCATTTGTGGTTGGAG-3′ | 121 |
| *PaKnottedL1* | 5′-ATTGGTGGGCTCGTCACTAC-3′ | 5′-AAACTGCATCTCCTCCGATG-3′ | 152 |
| *PaKnolleL1* | 5′-GGACGGGCTATGGTTACAGA-3′ | 5′-CTTGCGCCTCAACTATCACA-3′ | 130 |
| *PaUbi1* | 5′-AACTCCATCGCCTTCCTCTT-3′ | 5′-TGAAGCATGGCATCAATTTC-3′ | 101 |
| *PaCDKA1* | 5′-AGGAAATGCAGCATGGAAAC-3′ | 5′-TGTCGAGCTCCAAGGAGAAT-3′ | 373 |
| *PaCDKB1* | 5′-TTCTGCTTGGATCGACACAC-3′ | 5′-GCTTCCTTTGCAGAAATTCG-3′ | 319 |
| *PaCDKB2* | 5′-AGGGCATTGCATTCTGTCAT-3′ | 5′-AAATGTGGAGGAGCTGCTGT-3′ | 312 |
| *PaDP1* | 5′-AGGCAGAACGTGCTTCTCTC-3′ | 5′-TCGTTCGCAGAATCTCATTG-3′ | 302 |
| *18S rRNA* | 5′-TTAGGCCACGGAAGTTTGAG-3′ | 5′-ACACTTCACCGGACCATTCAA-3′ | 280 |

Supplementary Table S5. Primer pairs for RACE-PCR

| **Gene name** | **5′ RACE primer** | **3′ RACE primer** |
| --- | --- | --- |
| *PaCYCA2;1* | 5′-GGCATAGAGGCTGCACATTT-3′ |  |
|  | 5′-TTTCCCTCCACCATTACTGC-3′ |  |
| *PaCYCA2;2* | 5’-CCAATGTCCATCCAGCAAG-3’ |  |
|  | 5’-GGCATAGAGGCTGCACATTT-3’ |  |
| *PaCYCA2;3* | 5’-TCAACCAGCCAATCAATCAA-3’ | 5’-ATGGGACCATGCAAAAcAATA-3’ |
|  | 5’-GTAGAGGCTGCACATTTGAGG-3’ | 5’-TCAAATGTGCAGCCTCTACG-3’ |
| *PaCYCA3;1* | 5′-AAACACCCAAAAGCTGCAAT-3′ | 5′-GATTTTGGTGGATTGGTTGG-3′ |
|  | 5′-CCACTTCAACCAACCAATCC-3′ | 5′-GCCAAAGGTGGAGGATTTCT-3′ |
| *PaCYCA3;2* | 5’-GCCTTCAAGAACCTCCTCAG-3’ | 5’-CAGCTTATTGGGGTTTCAGC-3’ |
|  | 5’-CGCTTTCCATCTCAACAACC-3’ | 5’-CATGCTCATTGCCTCAAAGT-3’ |
| *PaCYCB1;2* | 5′-TGGATCCCACATGCAATCTT-3′ | 5′-TGGTCAATCTTCGCATAGCA-3′ |
|  | 5′-TGCAATCTTGCTACGAGCTG-3′ | 5′-AAATCCCCCGTCAAAAATTC-3′ |
| *PaCYCB2;1* | 5′-TTCGTTTCCAGATCCTCCAT-3′ |  |
| *PaCYCB3;1* | 5’-AATATGAAAATCTCCATTCCAAGC-3’ | 5’-TGTTTGGTCTTACCACACTTCTTC-3’ |
|  | 5’-GACCAATACGCTTCGCATCT-3’ | 5’-CCACACTTCTTCTGGCATCT-3’ |
|  | 5’- TGGGTTTCACATACCGTTCTC-3’ |  |
| *PaCYCD1;1* | 5’-GAAGGACAATGGTCCAGGAA-3’ | 5’-CGATGAGTCGATTGCTGTGT-3’ |
|  | 5’-GGGTACAATGGTTTCCTCCA-3’ |  |
| *PaCYCD1;2* | 5’-CCGTCGTCGGATAAAATGAT-3’ | 5’-CCCTGTTTCCAGATCTCCAG-3’ |
|  | 5’-GGAGAAAGGAGGTGGGAGAA-3’ | 5’-TAACCCCGAGTTTGTCTTCG-3’ |
| *PaCYCD1;3* | 5’-GGTGAAGGACTTCTGGGTGT-3’ |  |
|  | 5’-AATCTGGAGGTCGAGAAGCA-3’ |  |
| *PaCYCD2;1* | 5′-TGAACCTTCCGAATCCAATC-3′ |  |
|  | 5′-TAATCTCTCGCCGGAAGAAG-3′ |  |
| *PaCYCD2;2* | 5′-ATGGCTAAGGAAAGGCAGGT-3′ |  |
|  | 5′-TCGTGTGTGGAGAGAAATCG-3′ |  |
| *PaCYCD4;2* | 5′-CCAATCAATGGCATGTGTTC-3′ | 5′-GGCCAAGTTTGTTTTTGAGG-3′ |
|  | 5′-GGCATATGCTCTGCCTCTCT-3′ | 5′-TTTGCCATCCAGTCAATCAA-3′ |
| *PaCYCD5;1* | 5’-AGCCGAAGCATTCTTTCATC-3’ | 5’-AAGTCTTGGGCACGTCAGTT-3’ |
| *PaCYCD5;2* | 5’-ATAAAAGCTTCATCGCCCATT-3’ | 5’-AACTCCGTCACTCCATTTGC-3’ |
|  | 5’-GTACTAACCATGAGGTGATTGCAG-3’ | 5’-AGAACTGCTCGGGAAAGCTAC-3’ |
| *PaCYCD5;3* | 5’-CGGAGGAATCAGGTGAATTT-3’ | 5’-GAGCTCAGTGACTCCACTTCC-3’ |
|  | 5’-TCGATTCCCAAGTCATCAGA-3’ | 5’-TGGCAAGAACTTTTCAAAGGA-3’ |
| *PaCYCD5;4* | 5’-CTGAAGGGCGGTAAAACATC-3’ | 5’-TGGACACACTGGAATGGAAA-3’ |
|  | 5’-TTCCATTCCAGTGTGTCCAA-3’ |  |
| *PaE2F2* |  | 5′-TGAGGACGGGACACTTGATT-3′ |
|  |  | 5′-CTCCATGGGTCCTGTTGATT-3′ |
| *PaE2F3* |  | 5’-GGTTTGCGTTTTGCTTTGAG-3’ |
| *PaE2F4* | 5’-TTGATGCAGAAGGGAGATTTG-3’ | 5’-ACCTGGTGAAGATGACGAAGTT-3’ |
|  | 5’-CTATTGGACCCATGGTGCTT-3’ | 5’-CTGCCATGCTTTCAGAATCA-3’ |
|  | 5’-AGAGCATTGAGGGAGAGGAAG-3’ |  |
| *PaDP2* | 5’-TGGCCTTTGGATTAGTTTGG-3’ | 5’-GCTGAAAAGTGGGCTTCTTG-3’ |
|  | 5’-AGCAGCTGATAGTGGGCATC-3’ | 5’GGCTGCTGAAAATGCAGATAAAAAT-3’ |
